# *Akkermansia muciniphila*'s *nifJ* gene enhances colostrum sIgA synthesis by branched-chain amino acid degradation to branched short-chain fatty acids

**DOI:** 10.1080/19490976.2026.2620128

**Published:** 2026-01-29

**Authors:** Deyuan Wu, Fuyong Li, Wenyu Xiong, Zihao Huang, Kaidi Ma, Jun Huang, Sensen Cai, Jinping Deng, Jie Peng, Xiong Xia, Chengquan Tan

**Affiliations:** a Guangdong Provincial Key Laboratory of Animal Nutrition Control, National Engineering Research Center for Breeding Swine Industry, Institute of Subtropical Animal Nutrition and Feed, College of Animal Science, South China Agricultural University, Guangzhou, Guangdong, China; b Innovative Institute of Animal Healthy Breeding, College of Animal Science and Technology, Zhongkai University of Agriculture and Engineering, Guangzhou, Guangdong, China; c State Key Laboratory of Quantitative Synthetic Biology, Shenzhen Institute of Synthetic Biology, Shenzhen Institutes of Advanced Technology, Chinese Academy of Sciences, Shenzhen, Guangdong, China

**Keywords:** *Akkermansia muciniphila*, colostrum sIgA, *nifJ*, bacterial metabolism

## Abstract

Colostrum secretory immunoglobulin A (sIgA) confers the first immune defense line for neonates to adapt to the external environment, while gut microbes have received attention for their high reactivity with sIgA. Here, we report that high levels of sIgA in sow colostrum are associated with the enrichment of *Akkermansia muciniphila* and branched-chain amino acid (BCAA) metabolism in the intestine. Mechanistically, we demonstrate through mice models that *A. muciniphila*-derived *nifJ* mediates BCAA degradation to branched short-chain fatty acids, which further enhance transforming growth factor-β (TGF-β) and C-C motif chemokine ligand 28 (CCL28) expression in mammary tissues through the G protein-coupled receptor pathway, ultimately increasing sIgA content in colostrum. These findings establish a mechanistic link between the maternal gut microbiota and offspring immune development, highlighting the specific functional localization of the *nifJ* gene of *A. muciniphila*.

## Introduction

Unlike the microenvironment of the maternal uterus, after birth, the fetus is directly exposed to a wide variety of viruses, bacteria, fungi, and environmental antigens from the external world.^1^ B cells in the neonatal gut are typically not observed until ~4 weeks postpartum, suggesting that the infant immune system is functionally immature and heavily dependent on maternal-derived immunomodulatory factors to distinguish between commensal microbes and pathogens.^2^^,^^3^ These immunogens, including immunoglobulins (Igs), are primarily transferred via breast milk. Studies of human colostrum Ig composition have identified immunoglobulins A (IgA) as the predominant Ig with a central role in regulating mucosal humoral immunity.^4^ In the past decades, IgA has been extensively studied in the context of infectious diseases, such as *Salmonella* infections.^5^^,^^6^ Recent studies have confirmed that necrotizing enterocolitis is associated with increasing domination by *Enterobacteriaceae* in the IgA-unbound fraction of the microbiota, further highlighting the critical role of maternal secretory immunoglobulin A (sIgA) in breast milk in preventing experimental colitis.^2^ This was further supported by the report that mammary gland-derived sIgA selectively binds specific gut microorganisms and modulates the intestinal RORγt^+^ Treg population in offspring.^7^ Collectively, these findings indicate that sIgA plays the dual protective role of not only shielding the infant gut from pathogens but also actively shaping a balanced microbiota.

Since the specificity of colostrum sIgA reflects environmental antigens present in the gut, IgA-producing plasma cells residing in the mammary gland were hypothesized to be initially primed in the intestine.^8^ Indeed, IgA^+^ plasma cells are sensitized in the lymphoid nodes of intestinal mucosal tissues and Peyer's patches,^9^ and they would be transported to the mammary glands due to the upregulation of mucosal chemokine C–C motif chemokine ligand 28 (CCL28) expression by mammary epithelial cells during lactation.^10^ This mechanism was further supported by a study on the origin of IgA^+^ plasma cells in the mammary gland, where the mammary IgA antibody repertoire in lactating mice was shown to mirror that found in the intestine.^11^

IgA exhibits broad reactivity to microbes, underscoring the well-established role of microbiota in driving intestinal IgA responses.^12^ Previous studies have demonstrated that colonization with segmented filamentous bacteria (SFB) can significantly enhance intestinal IgA production, with notably higher intestinal IgA levels in mice colonized with SFB versus those colonized with *Clostridium* strains.^13^^,^^14^ This indicates that certain gut microbes can more effectively induce intestinal IgA production. However, in a study on the association of 53 human-derived microbial strains with gut immune responses, microbes that induce IgA production were found not to be necessarily phylogenetically related.^15^ Similarly, a recent study indicated that strain-level differences in *Bacteroides ovatus* exhibit varying capacities to induce IgA.^16^ These findings emphasize the importance of identifying microbial-derived bioactive factors, such as metabolites or cellular components.

Therefore, this study aimed to investigate the mechanisms by which the gut microbiota promotes IgA synthesis in colostrum, with a particular focus on identifying the active microbial components involved. Specifically, we demonstrated that *Akkermansia muciniphila* can significantly increase sIgA production in both maternal colostrum and caecum. Mechanistically, transforming growth factor-β (TGF-β) was regulated by *A. muciniphila*. Concurrently, the upregulation of homing chemokine CCL28 expression was observed in mammary tissues, facilitating plasma cell recruitment. Notably, *A. muciniphila* exhibits high expression of the branched-chain amino acid (BCAA) degradation pathway, a process facilitated by the pyruvate-ferredoxin oxidoreductase (PFOR) encoded by the *nifJ* gene. This metabolic activity ultimately generates branched short-chain fatty acids (BSCFAs), which act as signaling molecules via the G protein-coupled receptor 41 (GPR41) to increase sIgA synthesis.

## Materials and methods

### Animal model feeding and management

Because of their physiological similarity to humans, pigs have long been favored as a commonly used animal model in biomedical research of human pregnancy.^17^ Additionally, emerging evidence reveals the highest sequence homology between porcine IgA and human IgA among common animal models.^18^ For these reasons, this study utilized Duroc × Landrace × Yorkshire (DLY) ternary heterozygous primiparous sows as the experimental model. On day 65 of gestation, 50 pregnant sows with similar expected farrowing dates were enrolled in the trial. Information on sows is provided in Supplementary Table S1. All the experimental diets were formulated to meet or exceed the nutritional requirements for pregnant sows as recommended by the National Research Council (NRC, 2012). All procedures involving animals were carried out in accordance with the protocol approved by the Animal Ethics Committee of South China Agricultural University (Ethics approval number: 2022F239). All sows were housed in individual gestation stalls with free access to water. During late gestation, sows received 2.0 kg of feed per day administered in a single feeding at 05:30. The feeding regimen was gradually increased to 2.5 kg/d beginning 15 d before parturition. The environmental conditions were maintained within the thermoneutral zone through automated ventilation systems. On day 110 of gestation, fresh fecal samples were collected from each experimental sow via rectal massage and immediately stored at −80 °C for subsequent analysis. On the day of parturition, after cleaning the mammary glands with soapy water, colostrum was aseptically collected from the anterior, middle, and posterior mammary pairs. The colostrum samples collected from different mammary glands of each sow were mixed to eliminate differences in lactation sites. Finally, all the samples were stored at −80 °C for rapid preservation.

### Antibiotic (ABX) treatment

Briefly, 8-week-old C57BL/6J mice were obtained from SPFbiotech Co., Ltd. (Beijing, China) and then housed under controlled conditions (23 ± 3 °C, 70% ± 10% humidity) with 12-h light/dark cycles and free access to food and water. Following one week of acclimation, female, and male mice were paired at a 2:1 ratio for mating. Females with vaginal plugs observed the following morning were considered pregnant and individually housed. Females failing to conceive within 7 d were excluded from the study. Pregnant mice were randomly assigned to two experimental groups (*n* = 8 per group): the vehicle group and the ABX group. The antibiotic treatment protocol was adapted from a previous study with the following modifications:^19^ ABX group pregnant mice were treated with antibiotic cocktails composed of vancomycin (100 mg/kg), neomycin sulfate (200 mg/kg), metronidazole (200 mg/kg), and ampicillin (200 mg/kg) by oral gavage every 3 d from 0.5 d of embryonic age (E0.5d) to E19.5d.

### Treatment of *A. muciniphila* and its metabolites

*A. muciniphila* (ATCC BAA-835; purchased from Bosaibio) was cultured anaerobically (85% N_2_, 10% H_2_ and 5% CO_2_) in brain heart infusion broth supplemented with 0.5% porcine gastric mucin and 0.04% L-cysteine in anaerobic Glove Box (LAI-3, Shanghai Longyue Instrument Equipment Co., Ltd., China). Prior to treatment, 5-week-old female C57BL/6J mice were acclimatized for one week, followed by the depletion of the intestinal microbiota every 2 d for 2 weeks as previously described. Following microbiota depletion, the mice were randomly allocated into groups (*n* = 8 per group) and received treatments from week 8 until E19.5d. Specifically, mice were gavaged once every 2 d with phosphate buffered saline (PBS) solution (CON), live *A. muciniphila* (1 × 10^8^ CFU) (AKK), BSCFA mixture (isovaleric acid: 150 mM, isobutyric acid: 75 mM and 2-methylbutyric acid: 75 mM), inactivated *A. muciniphila* (1 × 10^8^ CFU) (KA), and BCAA mixture (0.75 mg/g body weight) (weight ratio, leucine:valine:isoleucine = 2:1:1; Sigma-Aldrich, St. Louis, MO, USA). For heat inactivation of *A. muciniphila,* live bacterial cultures (1 × 10^8^ CFU) were first centrifuged at 6000 × *g* for 15 min to collect the bacterial pellet, which was washed twice with sterile PBS, resuspended, and subsequently pasteurized at 75 °C for 30 min to ensure complete inactivation. Fresh transplant materials were prepared under sterile conditions daily at 18:00, 30 min prior to oral gavage. On the day of lactation (L0d), the female mice and offspring were euthanized, and cecum and mammary gland samples were collected from the female mice; colostrum samples were collected from white milk clots (white gastric contents) in the stomachs of the offspring.^20^

### Pharmacological blockade of GPR41

The 9-week-old pregnant female mice were randomly divided into three groups with six mice per group. The CON and the GPR41 inhibitor pertussis toxin (PTX) groups were gavaged separately with PBS and PTX solutions (5 µg/kg) every 2 d during gestation,^21^^,^^22^ and the PTX + BSCFA group was additionally gavaged with BSCFA mixture at E7.5d. Colostrum and cecum samples were collected after female mice and offspring were executed at L1d.

### Biochemical analysis

The contents of IgA, TGF-β, and IL-4 (Pig secretory IgA ELISA research kit_MM-36234O1, Pig TGF-β ELISA research kit_MM-0382O1 and Pig IL-4 ELISA Research KitMM-04190O1 MEIMIAN, China) in sow colostrum and IgA (Mouse secretory IgA ELISA research kit_MM-0430M2 MEIMIAN, China) in female mice colostrum were assayed as instructed by the manufacturers of the corresponding kits.

### Immunofluorescence staining

Mammary gland tissue sections were blocked with 5% bovine serum albumin (BSA) for 1 h and incubated with the antibodies sequentially. Primary antibodies were used at 1:250 dilution. The fluorescent secondary antibodies CoraLite488-conjugated Goat Anti-Rabbit IgG(H + L) (SA00013-2, Proteintech China) were used at 1:250 dilution, with antibodies shown in Supplementary Table S3. Finally, an LSM 700 confocal microscope (Zeiss, Germany) was used for image acquisition, and images were quantified with the Zen microscope software 2011, blue edition (Zeiss, Germany).

### SCFA analysis by gas chromatography with mass spectrometry (GC–MS)

The SCFA concentration in the contents of cecum was measured as previously reported.^23^ Briefly, the samples were thawed on ice, followed by placing 0.5 g of each sample into a centrifuge tube containing 1 mL of distilled water and vortexing for 1 min to homogenize the mixture. After being disrupted in an ultrasonic bath for 30 min, the samples were centrifuged at 13,000 rpm for 10 min, and the supernatant was collected. All the supernatant was poured into another 2 mL centrifuge tube, followed by adding 20 μL of 25% metaphosphoric acid and 0.25 g of anhydrous sodium sulfate and vortexing for 1 min to homogenize the mixture thoroughly. After adding 1 mL of methyl tert-butyl ether (operated in a fume hood), the mixture was vortexed for 5 min to homogenize thoroughly and then centrifuged at 13,000 rpm for 5 min to collect the upper layer of the methyl tert-butyl ether extract (operated in a fume hood). After filtration through a 0.22 μm microporous filter membrane, the filtrate was placed into a sample vial with a lined tube, followed by incubation at 80 °C for 20 min and standing for 48 h prior to the on-board assay. Finally, the sample was analyzed using a gas chromatography‒tandem mass spectrometry platform on a Shimadzu GCMS-TQ8040 triple quadrupole mass spectrometer (Shimadzu) equipped with a capillary column (BPX5) (SGE Analytical Science, Japan).

### Analysis of metabolites in caecum contents by liquid chromatography–mass spectrometry (LC–MS)

Briefly, frozen cecum content samples were thawed at 4 °C. For extraction of metabolites, each sample (~50 mg) was weighed, followed by adding 600 μL of methanol (chromatographic grade), homogenizing for 2 min, and breaking it by low-temperature sonication for 10 min and then for 30 min at −20 °C. After the samples were centrifuged at 4 °C and 14,500 rpm for 15 min, 200 μL of the supernatant was aspirated and dried in a vacuum centrifuge. Next, the supernatant was redissolved in 200 μL of 50% methanol, followed by shaking and mixing, and then centrifuged at 4 °C and 14,500 rpm for 15 min to collect the supernatant. After filtration through a 0.22 μm organic filter membrane, the filtrate was put into injection vials with inner liner caps for LC‒MS analysis. At the same time, the standards were diluted to different gradients and placed in sample vials, with standards including leucine (L0375000, Sigma), valine (V0030000, Sigma), and isoleucine (I0460000, Sigma). The content of metabolites in the standards and samples was detected by LC–MS (Thermo Fisher, USA), and then the metabolite content in each sample was quantified by Xcalibur (Thermo Fisher, USA).

### Quantification of specific bacterial species

Total bacterial DNA was extracted from the fecal samples using the fecal bacterial DNA extraction kit as instructed by the manufacturer (D3141, Magen, Shanghai). RT-qPCR was conducted using iScript One-Step RT-PCR Kit with SYBR Green (A0002, EZBioscience, China) on an ABI QuantStudio 6 Flex system (Applied Biosystems, Carlsbad, CA). Specific primers for total bacteria and specific bacterial species were synthesized by Sangon Biotech Co., Ltd. (Shanghai, China) and are shown in Supplementary Table S2. Standard curves were obtained by constructing standard plasmids containing 16S rRNA genes. The copy numbers of each target specific bacterial species were calculated using the corresponding standard curves, and the gene copy numbers were calculated using the equation of [DNA concentration (mg/mL) × 6.0233 × 10^23^ copies/mol]/[DNA size (bp) × 660 × 10^6^]. The relative abundance of *A. muciniphila* was the copy number ratio of individual specific bacterial species to total bacteria.

### Quantitative real-time PCR

RNA isolation and qPCR followed previously reported standard procedures.^24^ Briefly, total RNA from mammary glands (~50 mg) or cecum samples (~30 mg) was extracted with the reagent box of the total RNA kit as instructed by the manufacturer (EZB-RN001-plus, EZBioscience, China). The high-quality RNA was then reverse-transcribed using the Primer Script RT Kit (A0010CGQ, EZBioscience, China), followed by qPCR in ABI QuantStudio 6 Flex system (Applied Biosystems, Carlsbad, CA) using the iScript One-Step RT-PCR Kit with SYBR Green (A0002, EZBioscience, China), with the primers listed in Supplementary Table S2. Difference in transcript levels was quantified by normalization of each amplicon to 18S rRNA.

### Western blotting

Total proteins were extracted from cecum and mammary tissues as instructed by the manufacturer of the protein extraction kit (Beyotime, Beijing, China). A total of 10 μg protein was loaded and separated by SDS-PAGE gel electrophoresis, followed by transferring the proteins onto the polyvinylidene fluoride membranes (ISEQ00010, Merck Millipore, Merck KGaA, Germany). After being blocked with Tris-buffered saline with tween 20 containing 5% milk, the membranes were incubated first with the primary antibodies shown in Supplementary Table S3 and then with appropriate HRP-conjugated anti-rabbit IgG secondary antibody (AS014, Abclonal, China, 1:5000). Images were captured using the ChemiDoc MP system (Bio-Rad, Hercules, CA), and band densities were quantified using Image Lab software (Bio-Rad, Hercules, CA) and then normalized to the β-actin content.

### 16S rRNA gene amplicon sequencing, data processing, and analysis

Total genomic DNA from the sow fecal samples was extracted using the CTAB/SDS method, and the DNA concentration and purity were monitored on 1% agarose gels. According to the concentration, DNA was diluted to 1 μg/μL using sterile water. The highly variable V3-V4 region of the 16S rRNA gene was PCR-amplified from the extracted DNA using the primer pair 341F (5ʹ-CCTAYGGGRBGCASCAG-3ʹ) and 806R (5ʹ GGACTACHVGGGTWTCTAAT-3ʹ).^25^ The polymerase chain reaction (PCR) reactions were performed in a total volume of 50  μL, using 15  μL of Phusion High-Fidelity PCR Master Mix with GC BufferAmpliTaq Gold DNA Polymerase (New England Biolabs Inc., Beijing, China), 3 μL of primers, and 5–10 ng of DNA template. The PCR conditions were 98 °C for 1 min, followed by 30 cycles of 10 s at 98 °C, then 30 s at 50 °C and 30 s at 72 °C, and a final elongation step of 5 min at 72 °C. The final amplicon pool was evaluated using the Fragment Analyzer (Advanced Analytics Technologies, Ames, USA). The PCR products were also quantitatively assessed using a Qubit 2.0 Fluorometer (Life Technologies, USA) and diluted according to Illumina's standard protocol for Sequencing via Illumina MiSeq (Illumina Inc., USA).

The paired-end sequences of the 16S rRNA gene were assembled using FLASH (V1.2.11).^26^ Read quality checking and filtering were done using the standard operating procedure with QIIME (v1.17).^27^ Amplicon sequencing chimeras were removed using UCHIME (v4.2.40) algorithm,^28^ and the quality-filtered sequences were clustered into OTUs at a sequence similarity threshold of 97% using VSEARCH (v2.4.4).^29^ OTUs were assigned and identified to taxa using the SLIVA (v132), GreenGene (v13.8) and Unite (v7) reference libraries, and nonbacterial sequences (e.g. mitochondrial sequences) were removed.^30^ The rarefaction curve was produced in alpha_rarefaction.py script in QIIME, and alpha-diversity (Shannon, Chao1, Observed species or PD whole tree) was calculated using Mothur (v.1.48.0). Principal coordinate analysis (PCoA) was performed using the R package Vegan (v4.0.3) to summarize multidimensional clustering of microbial communities at the OTU level based on Bray–Curtis distances, and the ANOSIM test was selected to determine whether differences between groups were statistically significant.

Additionally, key taxa were identified as follows: (1) Differential abundance analysis of microbial taxa was performed using DESeq2 (v1.40.0) in R (v4.3.0). All *p*-values were adjusted for multiple testing using the Benjamini–Hochberg false discovery rate (FDR) correction, with an FDR < 0.05 was considered statistically significant. (2) The relationship between microbial communities and colostrum measurement indicators was analyzed by redundancy analysis (RDA) based on genus-level microbial abundance data using the vegan package (v2.6.4), with sample metadata and immune indicators (IgA, TGF-β, and IL-4) included as explanatory variables and data visualization implemented using ggplot2 (v3.4.2). (3) A Sankey diagram was drawn to visualize the multilevel flow relationships using the networkD3 package (v0.4) and the ggalluvial package (v0.12.5). Manhattan plot analysis was performed for visualization of the significance across taxonomic levels using qqman package (v0.1.9). (4) Classification models were built using the RandomForest package (v4.7.1.1), with the top 15 important feature taxa selected. Linear discriminant analysis effect size (LEfSe) was performed using the microbiomeMarker package (v1.6.0), with parameters set at the LDA score threshold > 2.0 and *p* < 0.05. (5) Microbial metabolic functions were predicted using PICRUSt2 (v2.5.2), with differential functional analysis performed via STAMP software (v2.1.3). (6) Specialized primary metabolic gene clusters relevant to the gut environment in our target bacteria (*A. muciniphila*) were systematically identified using the gutSMASH tool (version 1.0.0) based on its primary publication.^31^

### Preparation of an *Escherichia coli* strain deficient in *ydbK* gene

*E. coli* MG1655 strain DNA was extracted and used as a template to amplify the upper and lower homology arms required for *ydbK* knockdown, and the amplified fragments were seamlessly clonally ligated. After mixing and standing on ice for 30 min, the amplified products were transformed into *E. coli* DH5α receptor cells in LB/Amp/IPTG/xgal plate overnight at 37 °C, and a white clone strain named *ydbK*-ud-pUC19 was selected, which was used as a template for PCR amplification to obtain the repaired homologous arm. After activated culture of *E. coli* MG1655 bacteria at 37 °C, the monoclonal bacteria were inoculated into 5 mL of LB liquid medium. On the second day, 1% of the bacterial culture was transferred to 50 mL of LB liquid medium, and at an optical density (OD) of ~0.8, the bacterial body was collected by centrifugation, followed by three washes with 10% glycerol, and finally resuspending the bacterial body in 2 mL of 10% glycerol. After the extracted Cas9 plasmid was added to the prepared *E. coli* MG1655 receptor cells, the mixture was placed on ice for 5 min and then electroshocked at 2500 V. Subsequently, 1 mL of LB medium was added to fix the culture at 30 °C for 1 h to coat the Kan resistance plate. After adding the purified *ydbK*-ud fragment and prepared *ydbK* sgRNA plasmid to the electrotransformed receptor cells, the mixture was placed on ice for 5 min, followed by 2500 V electrotransformation, incubation for 1 h at 37 °C in 1 mL LB medium to coat the Kan resistance plate and screen for deletion mutant strains. Finally, the grown clones were identified by colony PCR, using the primers listed in Supplementary Table S4.

### *A. muciniphila*-derived *nifJ* gene is overexpressed in ∆*ydbK* *E. coli*

The pGEN vector framework was amplified using the pGEN plasmid as a template, and the *nifJ* fragment was amplified with *A. muciniphila* strain DNA as a template, using the primers shown in Supplementary Table S2. The amplified *nifJ* and pGEN fragments were seamlessly clonally ligated, and after mixing and standing on ice for 30 min, the amplified products were transformed into *E. coli* DH5α receptor cells. After being coated with LB-Amp plates and cultured overnight at 37 °C, the monoclonal strains were picked for identification. The correctly sequenced *ydbK*-ud-pUC19 plasmid was used as a template to amplify the *ydbK*-ud vector framework, and the correctly sequenced pGEN-*nifJ* plasmid was used as a template to amplify the *nifJ* gene fragment. After seamless ligation of the amplified *nifJ* fragment and the *ydbK*-ud vector fragment, the products were mixed well and placed on ice for 30 min before being transformed into *E. coli* DH5α receptor cells. After coating with LB-Amp plates and incubated overnight at 37 °C, the monoclonal strains were collected for identification. The *ydbK-nifJ*-pUC19 plasmid was used as a template to amplify the repair homology arm required for *ydbK-nifJ* knockdown replacement, and the amplified product was then purified for further use. After the purified *ydbK*-EM7-*nifJ*-ud fragment and the prepared *ydbK* sgRNA plasmid were added to *E. coli* MG1655 pCas electrotransformed receptor cells, the mixture was placed on ice for 5 min and electrotransformed at 2500 V, followed by the addition of 1 mL of LB medium, incubation at 37 °C for 1 h, and then coating with Kan plates to screen for *nifJ* overexpression strains. Finally, the grown monoclonal colonies were identified via PCR using the primers listed in Supplementary Table S4.

### Bacterial BCAA metabolism

To assess bacterial utilization of BCAA, 5 mM BCAA was added to the microbial culture media. Specifically, 50 mM mixed BCAA solution was filter-sterilized using a 0.22 μm membrane, followed by mixing microbial culture media with the prepared BCAA solution at a 9:1 (v/v) ratio to obtain medium containing 5 mM BCAA (with brain heart infusion broth supplemented with 0.5% porcine gastric mucin and 0.04% l-cysteine for *A. muciniphila* and LB medium for *E. coli*). The medium for *A. muciniphila* was prepared in an anaerobic glove box (LAI-3) and incubated for 12 h, and the control group received an equal volume of sterile PBS. Each strain was precultured under corresponding conditions, washed with PBS, and inoculated at an initial optical density (OD_600_ = 0.05) into medium supplemented or unsupplemented with BCAA. After incubated for 12 h under specified gas conditions (*A. muciniphila*: anaerobic, 37 °C; *E. coli*: aerobic, 37 °C), a portion of the culture was centrifuged (13,000 × g, 10 min, 4 °C), after which the supernatant was collected, filtered through a 0.22 μm membrane, and the resulting clear supernatant was stored at −80 °C for further analysis.

### Statistical analyses

Anderson–Darling and Levene's tests were used to determine the normality and homogeneity of data variance, respectively. The two-tailed Student's *t* test and the Mann‒Whitney *U* test were employed to evaluate the statistical significance of differences between groups. All the statistical analyses were performed using GraphPad Prism 9.5.1 software, and the results are presented as mean ± SEM unless otherwise specified. The results represent a single independent experiment, with the sample size for each experiment indicated in the figure legend. Data are determined to be statistically significant at *p* < 0.05 and expressed as *p < 0.05, **p < 0.01, ***p < 0.001, and ****p < 0.0001, with group comparisons shown in the corresponding figure. All other graphical representations are described in the figure legend.

## Results

### Colostrum sIgA synthesis is associated with maternal gut microbial composition

**Figure 1.**
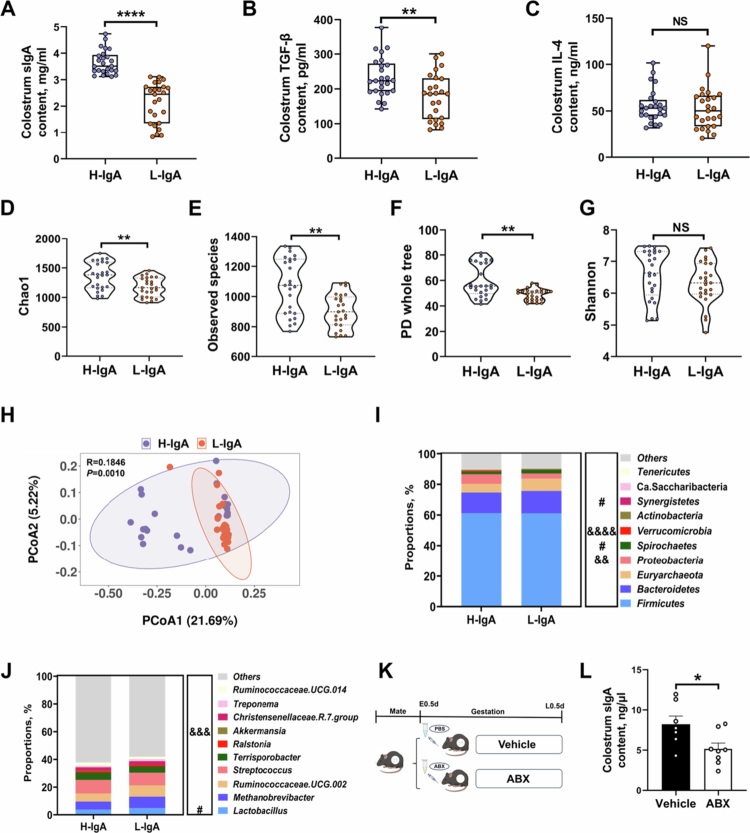
Gut microbes regulate sIgA levels in sow colostrum. (A) The sIgA content in sow colostrum (*n* = 25 sows for each group). (B) The TGF-β content in sow colostrum (*n* = 25 sows for each group). (C) The IL-4 content in sow colostrum (*n* = 25 sows for each group). (D–G) Alpha-diversity index (Chao1, observed species, PD whole tree, and Shannon indices) between H-IgA and L-IgA groups (*n* = 25 sows for each group). (H) Visualization of Bray‒Curtis-based principal coordinate analysis (PCoA) between H-IgA and L-IgA groups, with ANOSIM testing for differences (*n* = 25 sows for each group). (I, J) Average relative abundances of predominant taxa at the phylum and genus level (the Mann‒Whitney *U* test was used to assess differences, with “&” indicating significant enrichment in the H-IgA group and “#” indicating significant enrichment in the L-IgA group, *n* = 25 sows for each group). (K) Illustration of ABX-treated female mice experiment. (L) The sIgA content in female mice colostrum (*n* = 7 or 8 mice in each group). The data in (A–G and L) are presented as mean ± SEM, and an unpaired two-tailed *t* test was used to compare two groups (A–G and L). &, #,* indicate a significant difference at *p* < 0.05 (**^/&&^*p* < 0.01, ***^/&&&^*p* < 0.001, and ****^/&&&&^*p* < 0.0001); NS indicates not significant.

Colostrum samples were collected from 50 genetically homogeneous sows without nutritional intervention, and sIgA levels were measured (Figure 1A). The results fell within the physiological range of porcine colostrum sIgA.^32^^,^^33^ Based on sIgA content, sows were ranked, with the top 25 defined as the H-IgA group (average colostrum sIgA = 3.66 mg/mL) and the bottom 25 as the L-IgA group (average colostrum sIgA = 2.14 mg/mL). TGF-β and IL-4, which exert important influences on IgA production,^34^^,^^35^ were measured, and the results showed that H-IgA group sows were accompanied by abundant TGF-β, but with no significant changes in IL-4 (Figure 1B,C).

Considering the high cross-reactivity between sIgA and the gut microbiota,^36^ fecal samples from the 50 sows were subjected to 16S rRNA gene amplicon sequencing. As demonstrated by the rarefaction curve of the Observed_species index (Supplementary Figure S1A), the number of detected species plateaued with increasing sequencing depth, indicating sufficient coverage of microbial diversity and adequate data volume for downstream analyses. Additionally, in the L-IgA group, we found a significantly lower species diversity, mainly in Chao1, Observed_species, and PD whole tree indices (Figure 1D–F). The overall structural differences between the microbial communities of the two groups were visualized by Bray–Curtis-based PCoA, and the ANOSIM test demonstrated significant differences (Figure 1H, Supplementary Figure S1B). Microbial analysis revealed that at the phylum and genus levels, the relative abundances of p_*Proteobacteria*, p_*Verrucomicrobia*, and g_*Akkermansia* were significantly higher in the H-IgA group than in the L-IgA group (Figure 1I,J). Alterations in gut microbiota composition suggested that variations in colostrum sIgA levels might be influenced by intestinal microbes. We validated this hypothesis by using antibiotics cocktail to clear the intestinal microbiota (Figure 1K). Successful model establishment was confirmed by changes in the bacterial DNA content and 16S rRNA expression levels in murine cecum luminal samples (Supplementary Figure S1D,E). The results demonstrated that clearance of the gut microbiota in pregnant mice significantly reduced sIgA levels in maternal colostrum (Figure 1L). Collectively, our study provides further evidence for a strong association between colostrum sIgA content and the gut microbiota.

**Figure 2.**
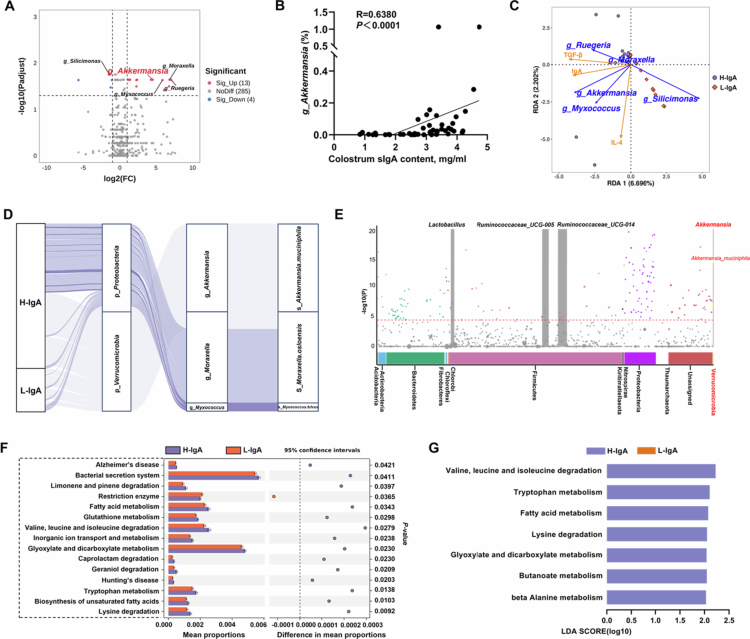
Screening of gut core microorganisms affecting the colostrum sIgA content. (A) The volcano plot generated from Deseq2 analysis of microbial groups with significant differences between the two groups. (B) Spearman correlation analysis of g_*Akkermansia* with colostrum sIgA. (C) Redundancy analysis (RDA) model used to assess the relationship of the core microbial community with colostrum sIgA and its regulators. (D) Sankey diagrams for the dynamic relationships between different taxonomic levels of microorganisms. (E) Manhattan plot of bacterial variability in the gut. (F) Relative abundance of KEGG pathways (top 15) in the gut as predicted by PICRUSt2. (G) Relative abundance of metabolism pathways (LDA > 2.0) in the gut as predicted by PICRUSt2 and analyzed by LEfSe. *n* = 25 sows for each group, and the Mann‒Whitney test was used to compare two groups. *p < 0.05.

Moreover, in-depth 16S rRNA gene amplicon sequencing data analysis identified g_*Akkermansia* as the key microorganism influencing colostrum sIgA production. Specifically, the volcano plot generated from Deseq2 analysis identified microbial groups with significant differences between the two groups. The most significantly different groups in the H-IgA group were g_*Akkermansia*, g_*Moraxella*, g_*Ruegeria*, and g_*Myxococcus*, in contrast to g_*Silicimonas* in the L-IgA group (Figure 2A). Spearman's correlation analysis revealed the strongest association between g_*Akkermansia* and colostrum IgA (Figure 2B, Supplementary Figure S2A–D). RDA analysis demonstrated a stronger covariation pattern between changes in g_*Akkermansia* abundance and the distribution of IgA and TGF-β concentrations in colostrum (Figure 2C). Additionally, random forest prediction and LEfSe analysis were performed to screen for characteristic markers. The results also revealed the highest mean decrease accuracy and LDA score for g_*Akkermansia* in the H-IgA group (Supplementary Figure S2E–G). Interestingly, in the LEfSe analysis results, g_*Lactobacillus* exhibited the highest LDA score in the L-IgA group (Supplementary Figure S2G). Previous studies have also found that supplementation with the human probiotic *Lactobacillus reuteri* is associated with a reduction in sIgA in breast milk.^37^ In summary, these results collectively underscore the pivotal role of g_*Akkermansia* in colostrum sIgA production. Additionally, species composition within the genus-level classification was analyzed by using a Sankey diagram to visualize multilevel flow relationships between taxonomic groups, and the results indicate that s_*Akkermansia.muciniphila* is the predominant member within the g_*Akkermansia* taxon (Figure 2D). Meanwhile, Manhattan plot analysis identified *A. muciniphila* as a significantly differentiated core microbe (Figure 2E).

Alterations in the gut microbial composition invariably drive functional remodeling of metabolic pathways, with downstream implications for host physiology. Using PICRUSt2, we predicted microbial KEGG metabolic functions and displayed the top 15 differentially abundant metabolic pathways in Figure 2F. Notably, the H-IgA group exhibited significant enrichment in the bacterial secretion system, glyoxylate and dicarboxylate metabolism, valine, leucine and isoleucine degradation, fatty acid metabolism, and tryptophan metabolism. Subsequent LEfSe analysis further evaluated the importance of these microbial metabolic pathways (Figure 2G), revealing that the highest LDA score was achieved by valine, leucine and isoleucine degradation. In summary, our results establish *A. muciniphila* as a core microbe in the H-IgA group and highlight its potential regulatory role in colostrum sIgA production, with the valine, leucine, and isoleucine degradation pathway probably serving as a critical functional link in this process.

### *A. muciniphila* enhances colostrum sIgA synthesis through T cell-independent pathway

**Figure 3.**
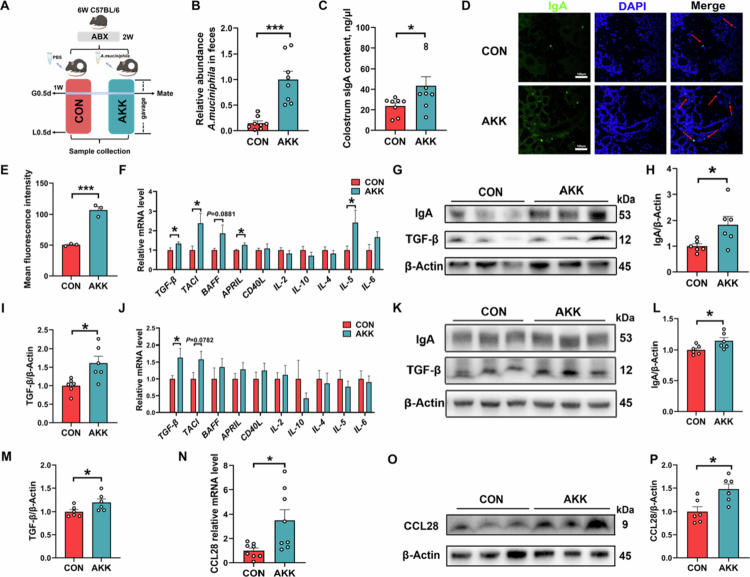
Oral gavage of *A. muciniphila* enhances colostrum sIgA production through a gut‒mammary axis. (A) Illustration of *A. muciniphila* gavage-treated female mice. (B) qPCR validation of *A. muciniphila* colonization in the gut (*n* = 8 mice for each group). (C) The sIgA content in female mice colostrum (*n* = 8 mice for each group). (D, E) Immunofluorescence and quantification of IgA in mammary tissues (*n* = 3 mice for each group). (F, J) The mRNA expression of IgA regulatory factors mediated through T cell-dependent and T cell-independent pathways in mammary and cecum tissues (*n* = 6‒8 mice for each group). (G, K) Representative immunoblots for protein expression of IgA and TGF-β in mammary and cecal tissues. (H, I, L, and M) Western blot analysis of the protein expression of IgA and TGF-β in mammary and cecal tissues (*n* = 6 mice for each group). (N) qPCR analysis of chemokine CCL28 levels in mammary tissues (*n* = 8 mice for each group). (O, P) Representative protein immunoblotting and quantitative results of CCL28 in mammary tissues (*n* = 6 mice for each group). The data in (B, C, E, F, H–J, L–N, and P) are presented as mean ± SEM. Unpaired two-tailed *t* test (B, E, F, H–J, L–N, and P) or Mann–Whitney test was used to compare two groups (C, F: TACI, BAFF, and IL-5, J: IL-10). *p < 0.05, ***p < 0.001, and 0.05 ≤ *p* ≤ 0.10 for a trend.

To investigate the regulatory effect of *A. muciniphila* on colostrum sIgA, we administered *A. muciniphila* to female mice via oral gavage, with detailed experimental information shown in Figure 3A. Successful cecal colonization of *A. muciniphila* was confirmed by qPCR analysis (Figure 3B). Further analysis revealed that oral gavage of *A. muciniphila* could significantly elevate sIgA levels in maternal colostrum (Figure 3C). Additionally, immunofluorescence staining of mammary tissues revealed significantly higher IgA levels in the AKK group than in the CON group (Figure 3D,E). As previously reported, the maturation and differentiation of B cells, along with IgA synthesis, are regulated by multiple cytokines.^38^^,^^39^ qPCR analysis of IgA-regulatory factors in mammary tissues showed that, compared to CON group, the AKK group was significantly higher in the expression of *TGF-β*, transmembrane activator and calcium-modulating cyclophilin-ligand interactor (*TACI*), a proliferation-inducing ligand (*APRIL*) and *IL-5*, with an uptrend in B cell-activating factor (*BAFF*) (Figure 3F). Notably, CD40L, the key mediator of T cell-dependent class-switch recombination (CSR), remained unchanged between the two groups. Consistently, western blot analysis also demonstrated significantly higher protein levels of both IgA and its pivotal regulator TGF-β in the AKK group than in the CON group (Figure 3G–I).

Since the majority of IgA⁺ plasma cells in mammary tissues originate from gut-associated lymphoid tissues (GALTs), we further examined IgA-regulatory factors in cecum samples. Compared with the CON group, the AKK group showed a significant increase in *TGF-β* mRNA expression (Figure 3J) and a corresponding increase in both IgA and TGF-β protein levels (Figure 3K–M). Additionally, enhanced IgA secretory responses were observed in both mammary and cecal tissues, indicating the existence of a gut‒mammary axis. The chemokine CCL28 mediates the homing migration of IgA antibody-secreting cells to mammary tissues, so we measured its level. As shown in Figure 3N, the AKK group exhibited significantly higher mRNA expression of CCL28, which was further confirmed by protein immunoblotting analysis (Figure 3O,P). The above results indicate that colonization of *A. muciniphila* in the caecum could stimulate the expression of IgA regulatory factors and promote IgA synthesis. Meanwhile, it upregulated the expression of CCL28 in the mammary glands, inducing the homing migration of IgA antibody-secreting cells to the mammary glands and increasing the IgA level in colostrum.

### *A. muciniphila* metabolizes BCAA to BSCFA

**Figure 4.**
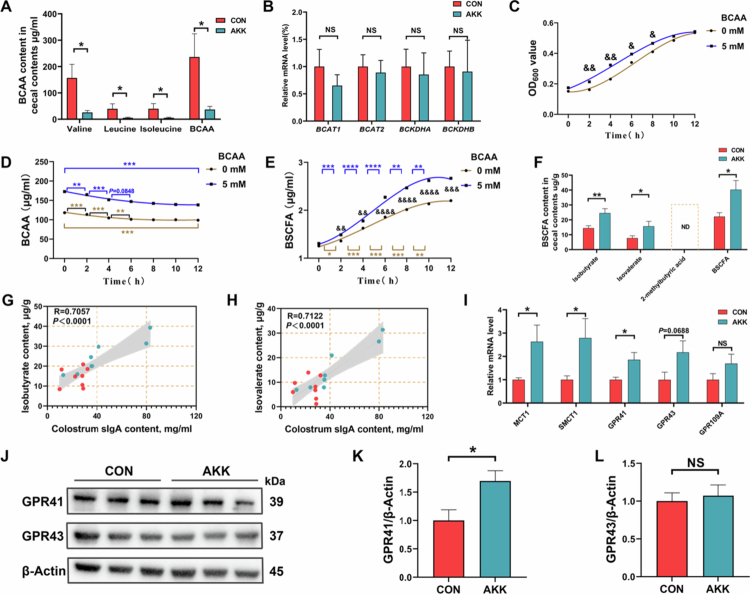
*A. muciniphila* is involved in BCAA degradation. (A). The branched-chain amino acid (BCAA) content in cecum contents (*n* = 8 mice for each group). (B) The mRNA expression of the rate-limiting enzyme of BCAA metabolic processes in cecal tissues (*n* = 8 mice for each group). (C) Additional BCAA supplementation promotes *A. muciniphila* growth (*n* = 2 for each group, with “&” for a significant difference at *p* < 0.05 in OD_600_ values of *A. muciniphila* cultured in medium supplemented with 0/5 mM BCAA at the same time point). (D, E) Time-course analysis of BCAA and BSCFA content in *A. muciniphila* culture supernatants (*n* = 2 or 3 for each group, with “&” for a significant difference at *p* < 0.05 in the BSCFA content of *A. muciniphila* cultured in medium supplemented with 0/5 mM BCAA at the same time point). (F) The BSCFA content in cecum contents (*n* = 8 mice for each group). (G, H) Pearson correlation analysis of isobutyrate and isovalerate with colostrum sIgA (*n* = 8 mice for each group). (I) The mRNA expression of BSCFA transporter proteins and downstream signaling receptors in cecum tissues (*n* = 7 or 8 mice for each group). (J) Representative immunoblots for protein expression of GPR41 and GPR43 in cecal tissues. (K and L) Western blot analysis of the protein levels of GPR41 and GPR43 in cecal tissues (*n* = 6 mice for each group). The data in (A–F, I, K, and L) are presented as mean ± SEM. Unpaired two-tailed *t* test (A–F, I, K, and L). ^&,^* indicate a significant difference at *p* < 0.05 (**^/&&^*p* < 0.01, ***^/&&&^*p* < 0.001, and ****^/&&&&^*p* < 0.0001), 0.05 ≤ *p* ≤ 0.10 for a trend, and NS for not significant.

BCAA can be converted into BSCFA in the intestine to regulate metabolism.^40^ In this study, PICRUSt2 prediction was performed, inferring the enrichment of the BCAA metabolic pathway in the intestines of H-IgA group sow (Figure 2F,G), and the oral administration of *A. muciniphila* was also shown to significantly reduce BCAA levels in the cecal contents of mice (Figure 4A). These results suggest that *A. muciniphila* may promote BCAA degradation in the intestine. To verify the above hypothesis, the expression of key rate-limiting enzymes involved in BCAA degradation in cecal tissue was first examined. As shown in Figure 4B, the two groups had no significant differences in mRNA expression levels of *BCAT1*, *BCAT2*, *BCKDHA*, or *BCKDHB*, thereby ruling out the potential confounding effects of maternal metabolic activity on the caecum amino acid pool. Subsequently, the ability of *A. muciniphila* in BCAA degradation was directly verified by adding 5 mM BCAA mixture to *A. muciniphila* medium. After 12 h of anaerobic cultivation, BCAA addition resulted in a more rapid increase in the number of *A. muciniphila* during the rapid growth phase (Figure 4C). Concurrently, we observed a decrease in BCAA levels in the culture medium and an increase in BSCFA content (Figure 4D,E). Unfortunately, one of the BSCFAs, 2-methylbutyric acid, failed to detected, probably because the undetectable concentration of 2-methylbutyric acid by our GC‒MS method. Although 2-methylbutyric acid was not detected, total BSCFAs showed significant differences in our statistical analysis. Collectively, our results indicated that *A. muciniphila* possesses the ability to degrade BCAA under *in vitro* cultivation conditions. Importantly, *in vivo* experiments revealed that oral administration of *A. muciniphila* could increase the BSCFA levels and the expression of related signal transduction proteins (particularly GPR41) in the cecal contents (Figure 4F,I–L). Furthermore, oral administration of *A. muciniphila* increased the levels of acetate, propionate, and valerate in the cecum contents (Supplementary Figure S3A), which is consistent with previous studies.^41^ However, Pearson correlation analysis showed only the significant positive correlation between BSCFA and colostrum sIgA levels (Figure 4G,H, Supplementary Figure S3B–H). These results indicate that *A. muciniphila* can degrade BCAA to BSCFA, which may be crucial for colostrum sIgA production.

### BSCFA mediates regulation of colostrum sIgA by *A. muciniphila*

**Figure 5.**
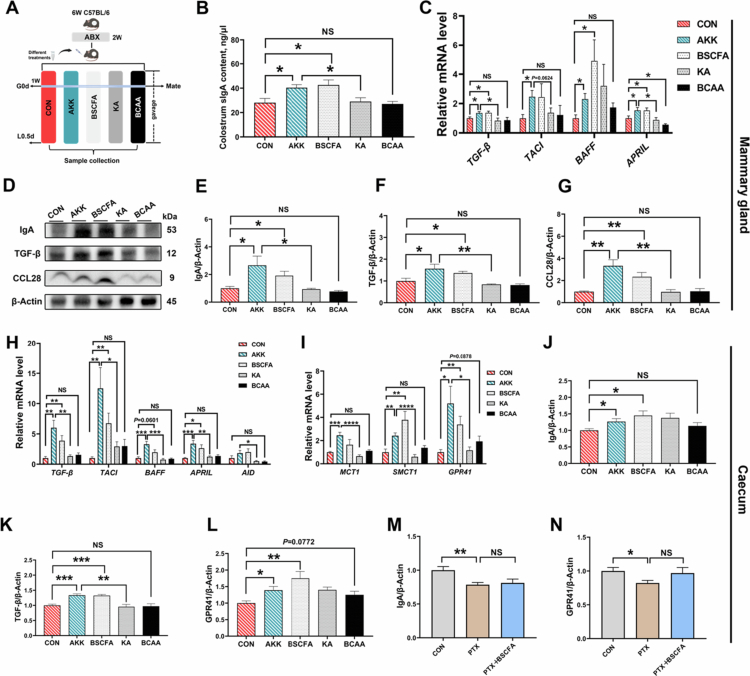
BSCFA mediates the regulation of colostrum sIgA by *A. muciniphila*. (A) Illustration of microorganisms and their metabolites treated by gavage in female mice. (B) The sIgA content in female mice colostrum (*n* = 8 mice for each group). (C, H) The mRNA expression of IgA regulatory factors in mammary and cecal tissues (*n* = 6–8 mice for each group). (D) Representative immunoblots for protein expression of IgA, TGF-β, and CCL28 in mammary tissues. (E–G) Western blot analysis of the protein levels of IgA, TGF-β, and CCL28 in mammary tissues (*n* = 6 mice for each group). (I) The relative mRNA expression levels of BSCFA signaling factors in cecal tissues (*n* = 8 mice for each group). (J–L) Western blot analysis of the protein levels of IgA, TGF-β, and GPR41 in cecal tissues (*n* = 6 mice for each group). (M, N) Western blot analysis of the protein levels of IgA and GPR41 in cecal tissues (*n* = 6 mice for each group). The data in (B, C, and E–N) are presented as mean ± SEM. Unpaired two-tailed *t* test was used to compare two groups (B, C, and E–N). *p < 0.05, **p < 0.01, ***p < 0.001, ****p < 0.0001, 0.05 ≤ *p* ≤ 0.10 for a trend, and NS for not significant.

The mediated effect of BSCFA on the regulation of colostrum sIgA by *A. muciniphila* was verified by gavaging female mice separately with PBS buffer, living *A. muciniphila*, BSCFA mixture, inactivated *A. muciniphila*, and BCAA mixture (Figure 5A). The success of the experimental treatments was confirmed by changes in BCAA and BSCFA concentrations in the cecum contents (Supplementary Figure S4A,B). The sIgA content in the colostrum was determined, and consistent with the AKK group, the BSCFA group was significantly higher than the CON group in the colostrum sIgA content, but with no significant differences between the BCAA and CON groups (Figure 5B). Similarly, the mRNA levels of IgA regulatory factors (*TGF-β*, *TACI*, *BAFF*, and *APRIL*) in mammary tissues were significantly higher in the BSCFA and AKK groups (Figure 5C). Additionally, the protein results showed that gavaging *A. muciniphila* and BSCFA could significantly up-regulate mammary tissue expression of IgA, TGF-β, and CCL28 (Figure 5D–G). Moreover, IgA responses in cecal tissues were also characterized, and the AKK and BSCFA groups showed significantly higher mRNA levels of the IgA regulators GPR41 and transporters of BSCFA in cecal tissues (Figure 5H,I), as well as higher protein levels of IgA, TGF-β, and GPR41 (Figure 5J–L). The above experimental results indicate that, as the metabolite of *A. muciniphila*, BSCFA plays an important role in colostrum sIgA production and GPR41 may act as a major signaling receptor to stimulate the subsequent IgA synthesis response.^42^ The regulatory role of GPR41 was verified in female mice (Supplementary Figure S4D). After gavaging the mice with PTX, we found a significant decrease in the colostrum sIgA content along with the caecum IgA protein levels, whereas BSCFA supplementation failed to reverse the decrease in sIgA (Supplementary Figure S4E,Figure 5M,N). These results highlight the special significance of GPR41 as a downstream signaling receptor of BSCFA in colostrum sIgA synthesis.

### The *nifJ* gene of *A. muciniphila* regulates BCAA catabolism

**Figure 6.**
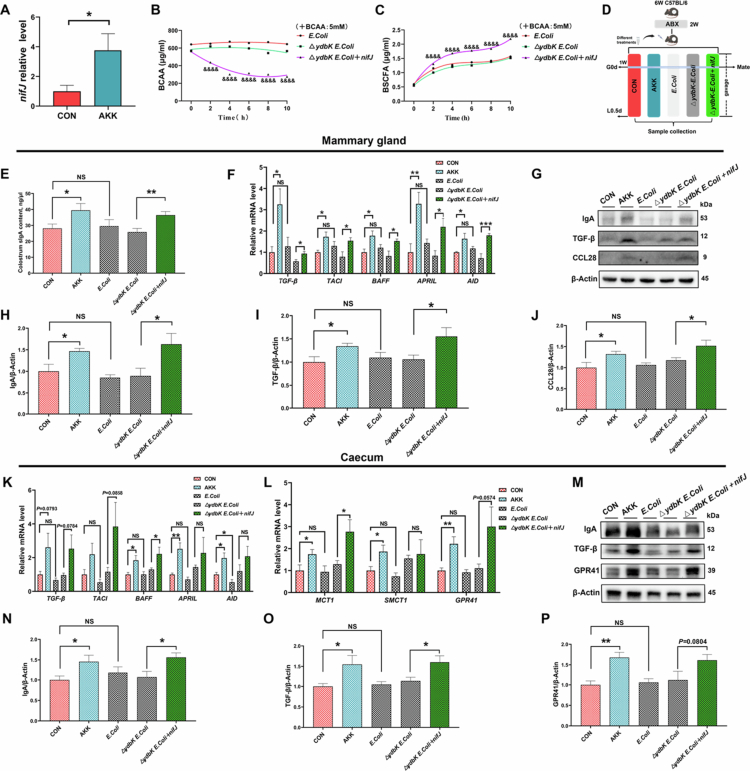
The *nifJ* gene of *A. muciniphila* mediates BCAA catabolism. (A) The *nifJ* gene expression in the caecum contents of female mice, with samples obtained from female mice as shown in Figure 3A (*n* = 6 mice for each group). (B, C) Time-course analysis of BCAA and BSCFA content in genetically engineered bacterial culture supernatants (with ^&^ for a significant difference at *p* < 0.05 between △*ydbK E. coli* and △*ydbK E. coli* + *nifJ*, *n* = 3 for each group). (D) Illustration of female mice gavaged separately with genetically engineered bacteria. (E) The sIgA content in female mice colostrum (*n* = 8 mice for each group). (F) Relative mRNA expression levels of regulators of the IgA response in mammary tissues (*n* = 8 mice for each group). (G–J) Western blot analysis of the protein levels of IgA, TGF-β, and GPR41 in mammary tissues (*n* = 6 mice for each group). (K) Relative mRNA expression levels of regulators of the IgA response in cecal tissues (*n* = 8 mice for each group). (L) Relative mRNA expression levels of BSCFA signaling factors in cecum tissues (*n* = 8 mice for each group). (M) Representative immunoblots of protein expression of IgA, TGF-β, and GPR41 in cecal tissues. (N–P) Western blot analysis of the protein levels of IgA, TGF-β, and GPR41 in cecal tissues (*n* = 6 mice for each group). The data in (A-C, E, F, H–L, and N–P) are presented as mean ± SEM, and unpaired two-tailed *t* tests was used to compare two groups (A–C, E, F, H–L, and N–P). &, * indicate a significant difference at *p* < 0.05 (&/**p < 0.01, &&&/***p < 0.001, &&&&/****p < 0.0001), 0.05 ≤ *p* ≤ 0.10 for a trend, and NS for not significant.

To understand the metabolic pathway of BCAA conversion into BSCFA by *A. muciniphila*, we predicted the metabolic gene function clusters of *A. muciniphila* (ATCC BAA-835) using gutSMASH. Previous studies have found that the BCAA degradation pathway of *Parabacteroides merdae* is regulated by the *porA* gene,^43^ which is a member of the PFOR family.^40^ Fortunately, the PFOR metabolic pathway was also identified in our metabolic gene function prediction of *A. muciniphila* (Supplementary Figure S5A,B), and its encoding gene was found to be *nifJ* by NCBI database comparison (Supplementary Figure S5C). In the cecum contents of *A. muciniphila*-gavaged mice (Figure 6A), we found a significant increase in the expression *of the nifJ* gene, which verified its presence in *A. muciniphila*.

We intended to construct a *nifJ* knockout strain of *A. muciniphila* but failed, probably because *nifJ* is an essential gene for this anaerobic bacterium,^44^ and its deletion could be lethal to *A. muciniphila*. The NCBI database revealed high sequence homology between the *ydbK* gene (NCBI Gene ID: 946587) in *E. coli* MG1655 and *A. muciniphila nifJ* (NCBI Gene ID: 60880315). We further analyzed the similarity of the proteins encoded by these two genes using the VectorBuilder tool (Yunzhou Biotech China), which revealed a similarity of 68.85%. Considering the considerable evolutionary distance between these two species on the phylogenetic tree, retaining 68.85% sequence identity suggests that these two genes are likely orthologs. Furthermore, as a facultative anaerobe, *E. coli* may potentially tolerate *ydbK* deletion without lethal consequences, so we constructed a *ydbK*-deficient bacterium of △*ydbK E. coli* and a *nifJ* backfill bacterium of △*ydbK E. coli* + *nifJ*. The success of the two genetically engineered bacterial constructs was confirmed by agarose gel electrophoresis (Supplementary Figure S6A–D). Next, we verified the action of both genetically engineered bacteria on BCAA catabolism, and compared to the original strain, *ydbK* deletion did not affect the action of *E. coli* on BCAA catabolism. However, backfilling the *nifJ* gene of *A. muciniphila* on top of *ydbK* deletion enabled *E. coli* to degrade BCAA significantly and increase BSCFA production. (Figure 6B,C).

Subsequently, we validated the role of genetically engineered bacteria in female mice (Figure 6D), and the success of bacterial colonization was confirmed (Supplementary Figure S6E,F). As shown by the metabolites detected in the cecal contents of female mice (Supplementary Figure S6G,H), compared with △*ydbK E. coli*, △*ydbK E. coli* + *nifJ* could significantly decrease the BCAA content and increase the BSCFA abundance, coupled with a significant increase in the colostrum sIgA content (Figure 6E). Furthermore, we observed significantly elevated levels of IgA and TGF-β proteins and mRNA, along with CCL28 protein levels, in mammary gland tissues from the Δ*ydbK E. coli* + *nifJ* group (Figure 6F‒J). Similarly, the △*ydbK E. coli* + *nifJ* group showed a significant increase in the mRNA levels of IgA regulatory factors, as well as GPR41 and transporters of BSCFA in cecal tissues (Figure 6K–P). In summary, these results confirm that the *nifJ* gene of *A. muciniphila* manipulates the metabolic process from BCAA to BSCFA.

## Discussion

The intrauterine environment has long been regarded as sterile, allowing fetal development without microbial challenges. However, during parturition, newborns suffer abrupt exposure to environmental microbes and potential pathogens. Over the past few decades, extensive research has demonstrated the pivotal role of sIgA in establishing and maintaining early immune defenses during infancy.^45^^,^^46^ Studies on early childhood allergic symptoms have revealed differences in bacterial recognition patterns of IgA within the fecal microbiota between healthy children and those with allergies. For instance, gut commensals such as *Faecalibacterium* and *Bacteroides* predominantly exist in IgA-coated forms in healthy children, whereas they remain unbound to IgA in children with allergic symptoms.^47^ Overall, sIgA acquired through colostrum ingestion serves as the first immunological defense line against pathogenic invasion in neonates.

In recent years, a variety of nutritional intervention strategies or means of gut microbial colonization have been reported to modulate sIgA production. For example, glutamine supplementation in mice for 14 d was found to decrease the abundance of *Firmicutes* in the jejunum, enhancing the production of sIgA in the mouse intestine.^48^ However, when these modulation modalities were summarized, most of them were found to target sIgA synthesis within the intestinal mucosa. To the best of our knowledge, few researchers have paid attention to the correlation between microorganisms and colostrum sIgA. In this study, we explored the microbial regulation of colostrum sIgA synthesis using pregnant sows as a physiological model.

There are numerous reports on the regulatory factors affecting IgA synthesis, and an increase in IgA content by adding TGF-β to lipopolysaccharide (LPS)-stimulated mouse B-cell cultures was reported decades ago.^49^ Consistent with these studies, we also found a significant increase in TGF-β in colostrum from H-IgA group sows. The homeostasis of the intestinal microbiota plays an indispensable role in the healthy development of organisms.^50^ For instance, gestational diabetes could disturb the intestinal microbiota of mice, allowing bacterial components (such as LPS) to enter the placenta through the circulation, triggering placental inflammation and endangering the health of newborns.^51^ In this study, L-IgA sows exhibited intestinal dysbiosis with reduced microbial diversity, and their intestinal microbial composition also differed significantly from that of H-IgA sows, as evidenced by a lower abundance of *Verrucomicrobia*. The sIgA content reduction in colostrum after antibiotic intervention in pregnant mice also aptly illustrates the critical role of the healthy flora structure in colostrum sIgA production.

Microbial communities able to coexist in mutual benefit with their hosts or maintain a dynamic equilibrium typically exhibit a high taxonomic diversity, a high microbial gene richness, and a stable core microbial community.^52^ For example, the two major phyla *Bacteroidetes* and *Firmicutes* make up the majority of gut bacteria, and their disturbed proportions may contribute to metabolic syndromes such as obesity and diabetes. Additionally, certain bacteria also contribute to host physiology through unique metabolic capabilities.^53^ The presence of *Akkermansia* was found to be positively correlated with IgA levels in a study on the association between the gut flora and immunity in healthy middle-aged and elderly people in Southwest China.^54^ This finding is consistent with the above analyzed results. For the intestinal microbiota of sows from both groups, a high abundance of *Akkermansia* was detected in fecal samples from H-IgA sows, with a significant positive correlation between the abundance of *Akkermansia* and the levels of sIgA and TGF-β in colostrum. These findings suggest that the colonization of *Akkermansia* in the intestine and the production of sIgA in colostrum are closely and intrinsically linked.

Studies on female mice gave us feedback on the way *A. muciniphila* promotes colostrum sIgA. Similar to the results in sows, *A. muciniphila* gavage increased colostrum sIgA levels in female mice and upregulated the TGF-β mRNA expression and protein levels in mammary tissue. TGF-β activation signaling can induce germline C_H_ gene transcription,^55^ a process that confers specificity to CSR and biases CSR results toward IgA production.^56^ At this process point, B cells produce IgA responses to commensal bacteria through both T cell-dependent and T cell-independent pathways, facilitating the establishment of reciprocal host-microbiota interactions.^57^ In this study, the CD40L gene, which is necessary for the T cell-dependent pathway, showed no upregulation in mammary tissues, while a significant increase was observed in the mRNA expression of stimulatory signals of the T cell-independent pathway, such as *BAFF*, *APRIL*, and *TACI*. This suggests that *A. muciniphila* can induce the expression of TGF-β and contributes to the development of IgA-specific CSR through the T cell-independent pathway, ultimately enabling an increase of sIgA in colostrum.

Early studies of sIgA specificity against enteric pathogens showed that IgA^+^ plasma cells in the mammary gland originate from GALT, emphasizing the existence of the enteromammary axis. Specifically, IgA^+^ plasma cells in mesenteric lymph node (MLN) migrate to the mammary gland during specific physiological periods in the mother^58^ and subsequently secrete IgA into colostrum, a process that requires the expression of the chemokine CCL28. Previous studies have also found that the amount of CCL28 mRNA is upregulated in the mammary gland of sows during late pregnancy and lactation.^59^^,^^60^ Therefore, we measured the gene expression and protein expression levels of IgA regulatory factors in mouse cecal tissues and obtained results similar to those in mammary tissues. Additionally, this study also found that the mRNA and protein expression levels of CCL28 varied significantly in mammary tissues. All these observations suggest that *A. muciniphila* gavage in mice can stimulate intestinal IgA-specific CSR while increasing the gene expression of CCL28 in mammary glands and inducing the aggregation of intestinal IgA^+^ secretory cells in mammary tissues.

*A. muciniphila* has been reported to induce brown adipose tissue uncoupling protein 1 and systemic glucagon-like peptide-1 secretion by secreting P9 protein to increase high-fat diet-induced thermogenesis in C57BL/6J mice.^61^ Moreover, small molecules or bioactive compounds secreted by gut microbes have been reported to be more beneficial to host health than the gut microbes themselves.^61^^,^^62^ These reports encouraged us to further explore the metabolites of *A. muciniphila*, and based on the metabolic pathway prediction results of bacterial 16S rRNA, we found that *A. muciniphila* gavage could reduce BCAA levels in cecum contents. After excluding the interference with the physiological metabolism of amino acids in mice, we confirmed that *A. muciniphila* can degrade BCAA to BSCFA and BSCFA are involved in the regulatory process of colostrum sIgA. Previous studies have shown that SCFA are produced during gut microbiota fermentation, which provide fuel for B cells via the SCFA receptor (G protein-coupled receptor) and promote IgA production.^63^^,^^64^ In the present study, BSCFA were found to upregulate the expression of GPR41 in the gut, and GPR41 has been shown to regulate TGF-β expression through its Gα_i/o_ subunits in a previous study.^65^ PTX has been reported to be a multisubunit toxin that binds most mammalian cells and specifically targets G proteins, thus able to inhibit G protein-coupled signaling pathways.^66^ Here, we used PTX-treated female mice to confirm the binding specificity and functional interaction between BSCFA and GPR41. Our results highlight that the metabolic product of BSCFA from *A. muciniphila* can stimulate TGF-β expression via the GPR41 signaling pathway to increase sIgA synthesis.

Previous studies have identified *P. merdae* as a gut commensal bacterium to convert BCAA to BSCFA, primarily through its *porA* gene-encoded activity.^43^ *PorA*, a member of the PFOR superfamily, catalyzes an unconventional pathway for BSCFA production by metabolizing branched-chain *α*-keto acids derived from BCAA.^67^ We found that *A. muciniphila*'s *nifJ* can also encode PFOR, and although *nifJ* knockout proved lethal to *A. muciniphila*, we discovered its functional analog *ydbK* in *E. coli*. Through *in vitro* cultivation of engineered strains and *in vivo* murine experiments, we conclusively demonstrated that *A. muciniphila*'s *nifJ* mediates BCAA degradation.

However, this study still has certain limitations. Although we found that the *nifJ* gene plays a dominant role in this process, the lack of purification and validation of the protein encoded by the *nifJ* gene means that the detailed mechanism by which *A. muiniphila* metabolizes BCAA and produces BSCFA remains unclear. Additionally, when its function was validated via gavage administration of *A. muciniphila*, we employed ABX mice instead of a germ-free mouse model. It must be acknowledged that ABX mice are not completely true germ-free mice, and the persuasiveness of our results could be undoubtedly enhanced by using germ-free mice.

**Figure 7.**
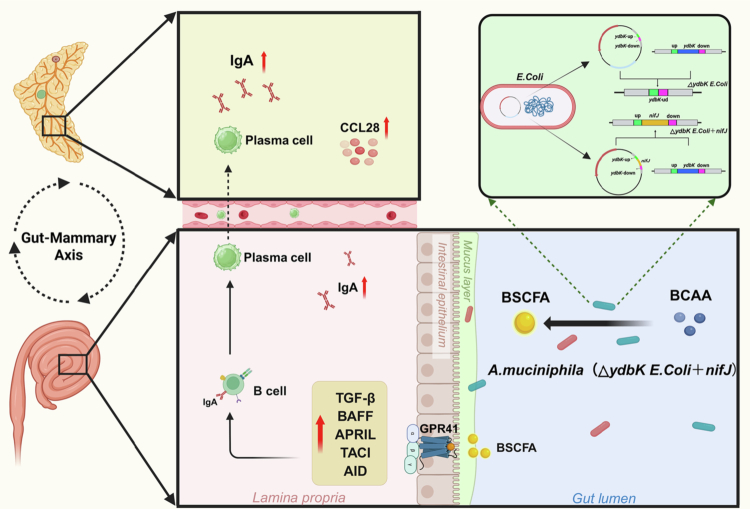
A working model illustrating the role of *A. muciniphila*-derived *nifJ* in colostrum sIgA biosynthesis. *A. muciniphila* utilizes its *nifJ*-encoded PFOR to catabolize BCAA in the host intestine and generate BSCFA. These BSCFA activate the GPR41 receptor to stimulate intestinal IgA CSR, and then the CCL28-mediated homing migration of IgA^+^ plasmablasts to mammary tissues, ultimately increasing sIgA production in colostrum.

Overall, as shown in Figure 7, this study suggests that colostrum sIgA synthesis is regulated by *A. muciniphila*. Specifically, *A. muciniphila* degrades BCAA to BSCFA in the intestine, which stimulates the IgA CSR response via its downstream GPR41 receptor and homing migration to mammary tissues in the presence of CCL28, thereby promoting colostrum sIgA production. In this study, we dissected the function of *A. muciniphila* and found for the first time the functional localization of the *nifJ* gene in *A. muciniphila*.

## Supplementary Material

clean_Supplementary_data.docx clean_Supplementary_data.docx

## Data Availability

Raw sequence data from all 16S rRNA gene sequencing experiments have been deposited in the Genome Sequence Archive (GSA) in National Genomics Data Center, China National Center for Bioinformation/Beijing Institute of Genomics, Chinese Academy of Sciences (GSA Accession number: CRA026948, PRJCA040863). Additional information and materials will be available upon reasonable request.
