# Supplementary material for: Akkermansia muciniphila's nifJ gene enhances colostrum sIgA synthesis by branched-chain amino acid degradation to branched short-chain fatty acids
Source: Gut Microbes. 2026 Jan 29;18(1):2620128. doi: 10.1080/19490976.2026.2620128 (PMC12867394; doi:10.1080/19490976.2026.2620128)
Supplement: clean_Supplementary_data.docx [file KGMI_A_2620128_SM4180.docx]

***Akkermansia muciniphila*’s *nifJ* gene enhances colostrum sIgA synthesis by branched-chain amino acid degradation to branched short-chain fatty acids**

Deyuan Wu^a^, Fuyong Li^a^, Wenyu Xiong^a^, Zihao Huang^a^, Kaidi Ma^a^, Jun Huang^a^, Sensen Cai^a^, Jie Peng^b *^, Xiong Xia^c *^, Chengquan Tan^a, *^

^a^Guangdong Provincial Key Laboratory of Animal Nutrition Control, National Engineering Research Center for Breeding Swine Industry, Institute of Subtropical Animal Nutrition and Feed, College of Animal Science, South China Agricultural University, Guangzhou, 510642, China.

^b^Innovative Institute of Animal Healthy Breeding, College of Animal Science and Technology, Zhongkai University of Agriculture and Engineering, Guangzhou, Guangdong, 510225, China.

^c^State Key Laboratory of Quantitative Synthetic Biology, Shenzhen Institute of Synthetic Biology, Shenzhen Institutes of Advanced Technology, Chinese Academy of Sciences, Shenzhen 518055, China.

*Corresponding author: [pengjie@zhku.edu.cn](mailto:pengjie@zhku.edu.cn); [x.xia@siat.ac.cn](mailto:x.xia@siat.ac.cn); tanchengquan@scau.edu.cn.


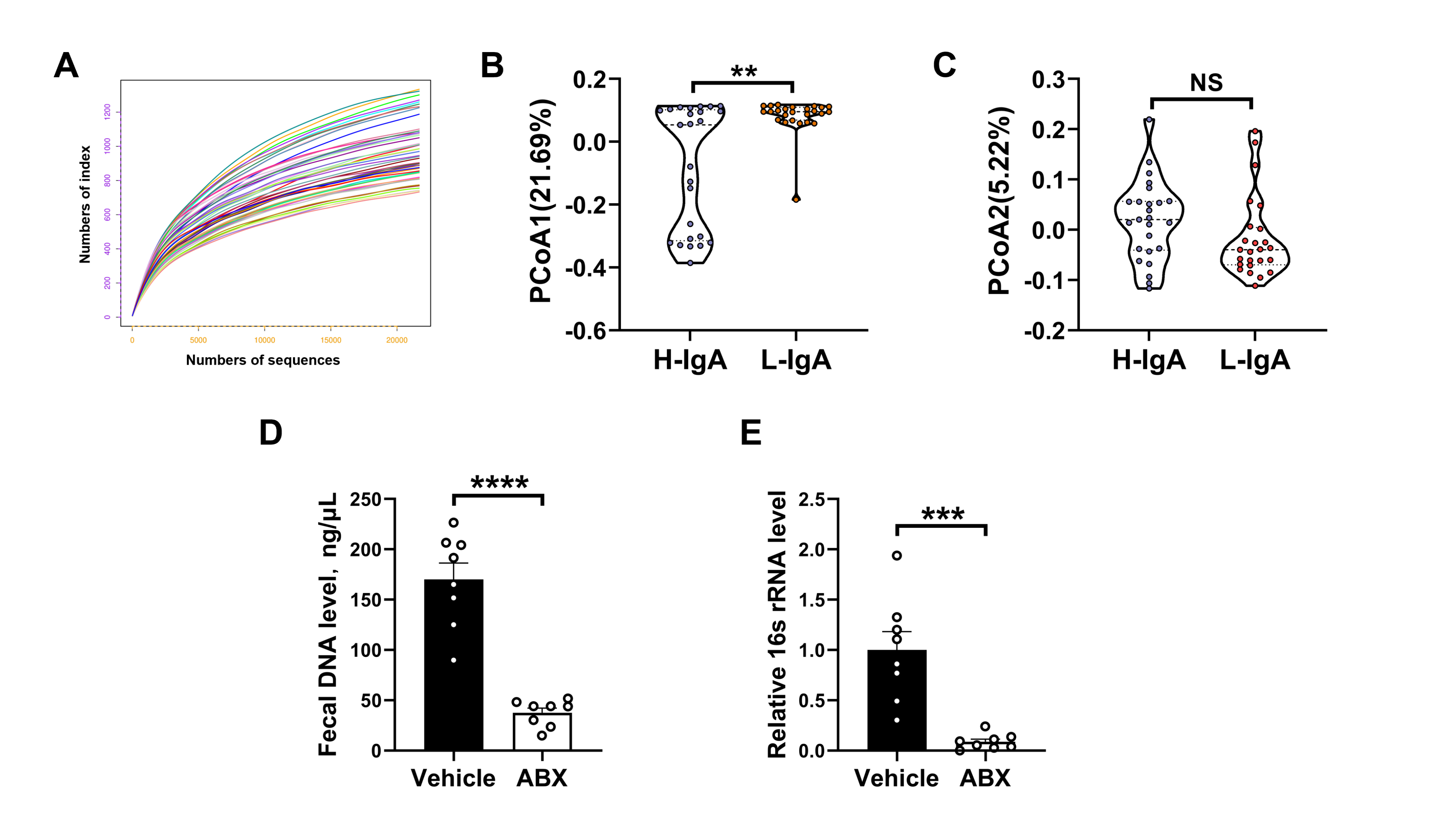


**Supplementary Figure S1. Quality assessment of 16S rRNA gene sequencing data and validation of gut microbiota depletion in mice.** (A) The rarefaction curves of the Observed_species index (*n* = 25 sows for each group). (B-C) Spatial distribution of sample locations in PCoA (*n* = 25 sows for each group). (D) Bacterial DNA concentration in fecal samples of ABX-treated mice (*n* = 8 mice for each group). (E) Bacterial 16s rRNA levels in fecal samples of ABX-treated mice (*n* = 8 mice for each group). Data in (B-E) are presented as mean ± SEM. Unpaired two-tailed t-test (D-E) or Mann-Whitney test was used to compare the two groups (B-C). ^**^ *p* < 0.01, ^***^ *p* < 0.001, and NS for not significant.


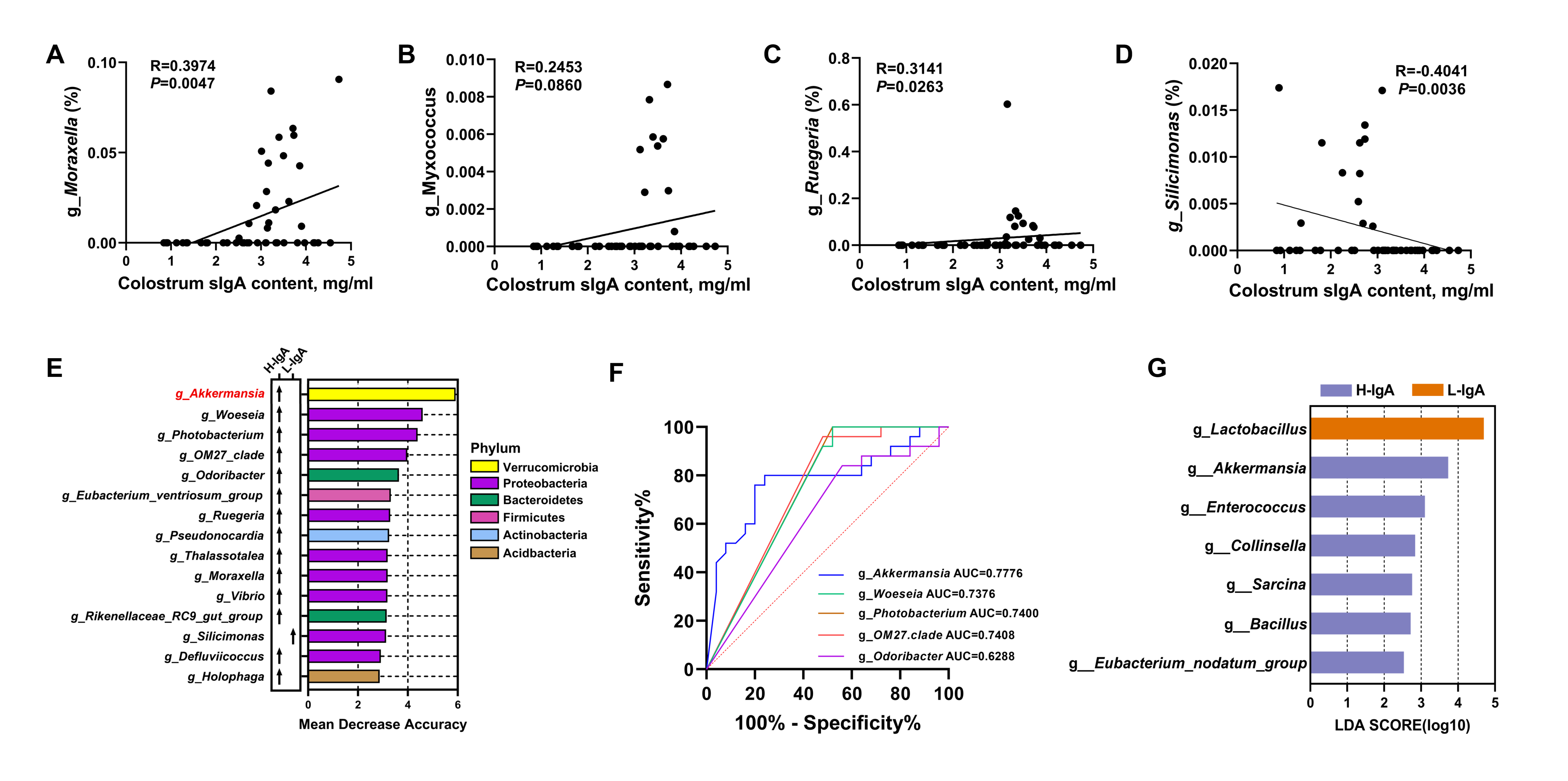


**Supplementary Figure S2. Gut microbiota is associated with colostrum sIgA content.** (A-D) Spearman correlation analysis of g_*Moraxella*, g_*Myxococcus*, g_*Ruegeria* and g_*Silicimonas* with colostrum sIgA (*n* = 25 sows for each group). (E) Random forest analysis for screening intergroup differences in bacterial genera. (F) ROC curve of the random forest model. (G) LEfSe analysis for identifying biomarkers.


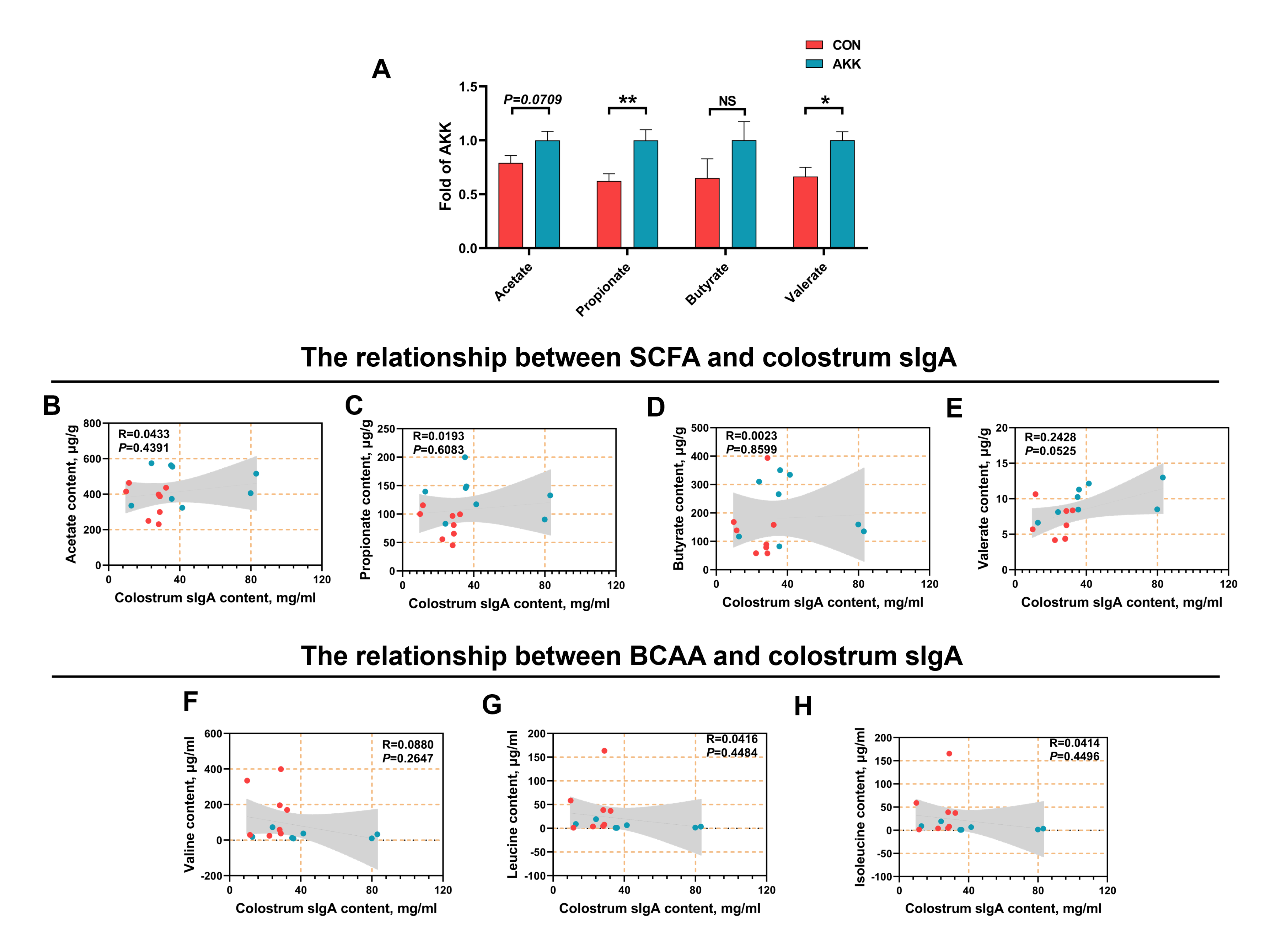


**Supplementary Figure S3. Correlation analysis between microbial metabolites and colostrum sIgA content.** (A) The SCFA content in caecum contents (Fold of AKK, *n* = 8 mice for each group). (B-E) The relationship between SCFA and colostrum sIgA (*n* = 8 mice for each group). (F-H) The relationship between BCAA and colostrum sIgA (*n* = 8 mice for each group). Data in (A) are presented as mean ± SEM and unpaired two-tailed t-test was used to compare the two groups (A). ^*^ *p* < 0.05, ^**^ *p* < 0.01, 0.05 ≤ *p* ≤ 0.10 for a trend, and NS for not significant.


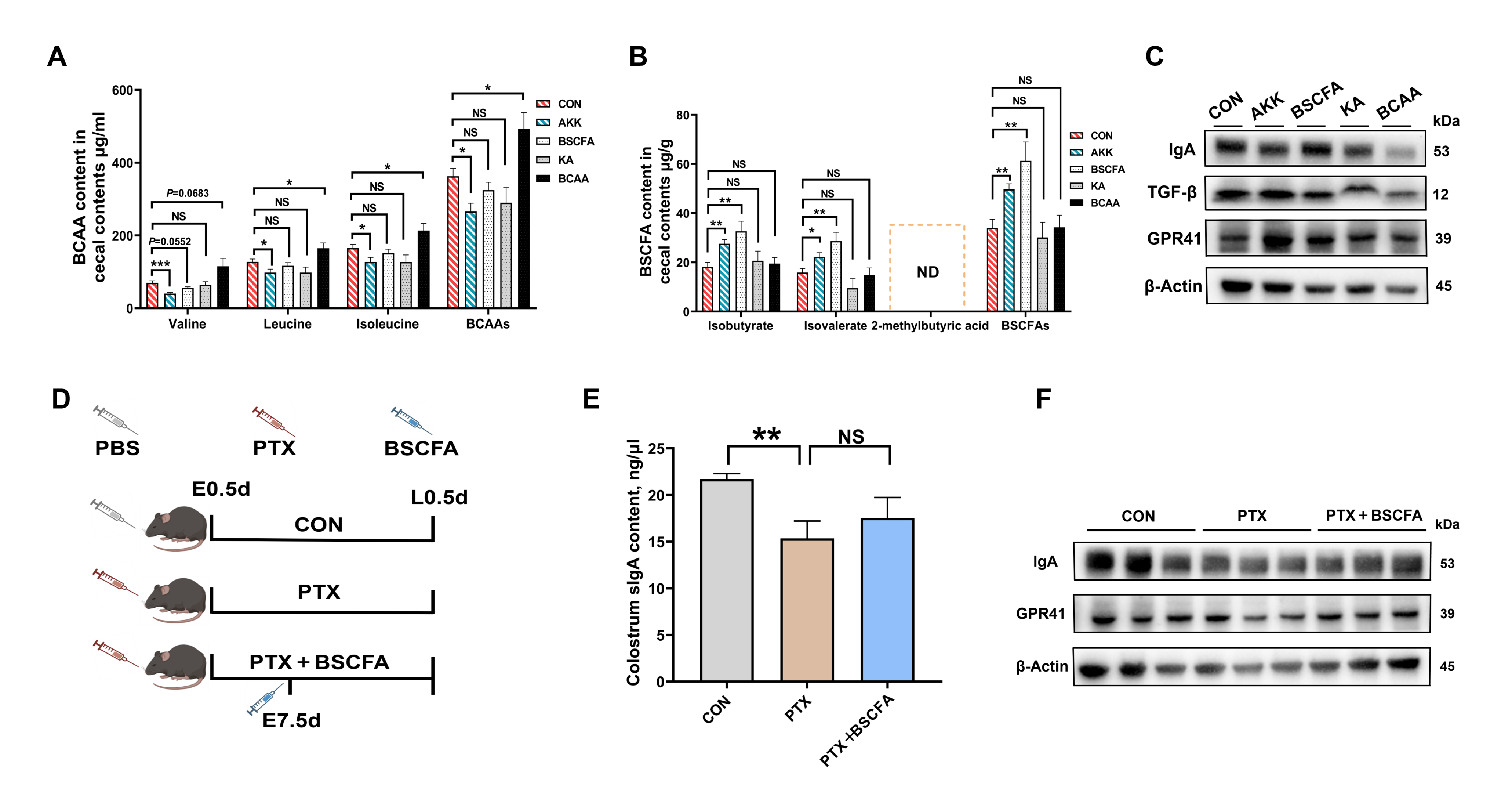


**Supplementary Figure S4.** **GPR41 mediates the regulatory effects of BSCFA on sIgA synthesis.** (A-B) The BCAA and BSCFA content in caecum contents of female mice (*n* = 8 mice for each group). (C) Representative immunoblots for protein expression of IgA, TGF-β and GPR41 in caecum tissues. (D) Illustration of GPR41 inhibitor-treated female mice. (E) The sIgA content in female mice colostrum (*n* = 6 mice for each group). (F) Representative immunoblots for protein expression of IgA and GPR41 in caecum tissues. Data in (A-B and E) are presented as mean ± SEM, and unpaired two-tailed t-test was used to compare the two groups (A-B and E). ^*^ *p* < 0.05, ^**^ *p* < 0.01, ^***^ *p* < 0.001, 0.05 ≤ *p* ≤ 0.10 for a trend, and NS for not significant.


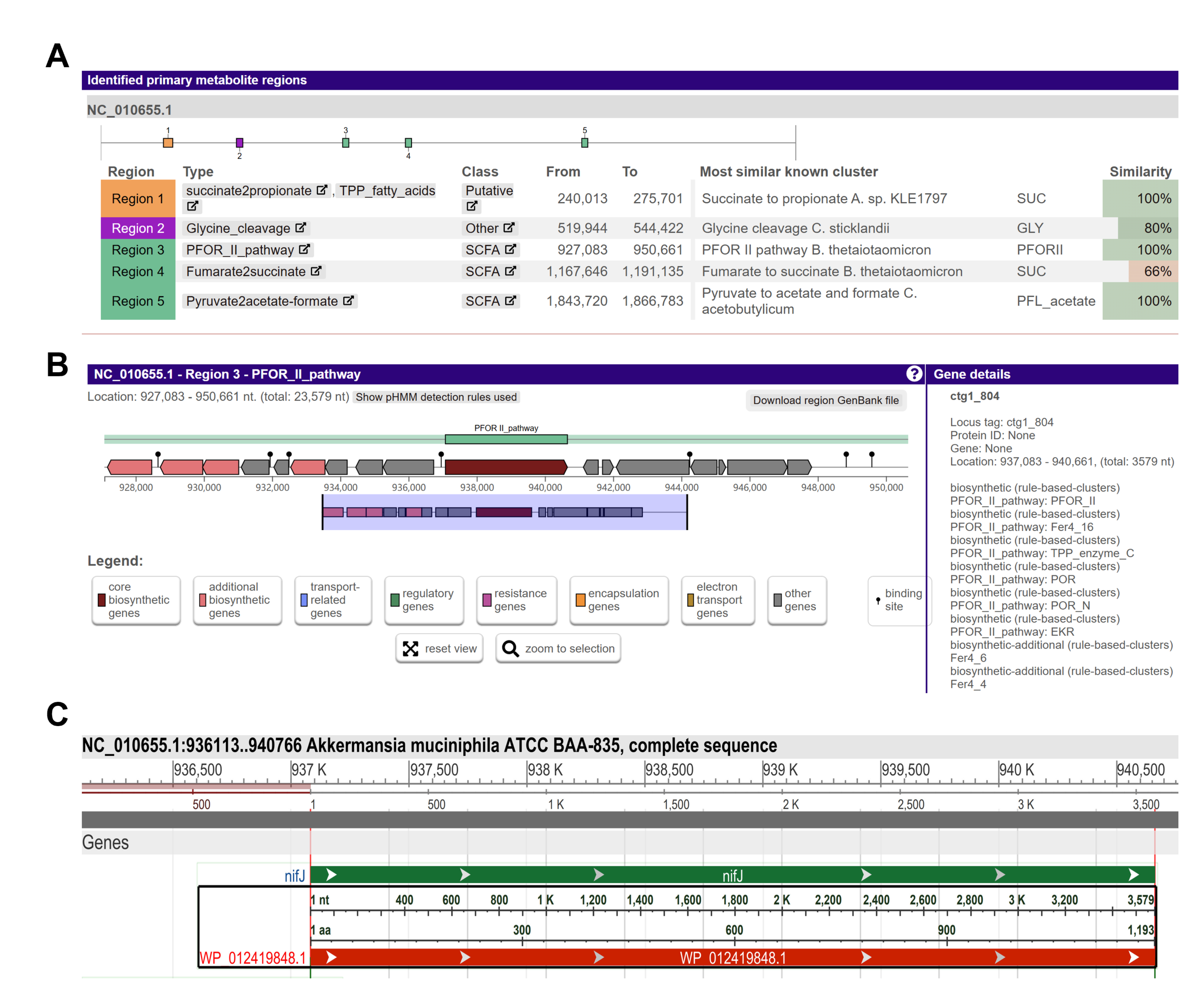


**Supplementary Figure S5.** **The *nifJ* gene in *A.muciniphila* encodes pyruvate-ferredoxin oxidoreductase (PFOR).** (A) gutSMASA prediction of metabolic functional gene clusters in *A.muciniphila*. (B) PFOR metabolic pathway in *A.muciniphila*. (C) NCBI database annotation of gene fragments encoding PFOR.


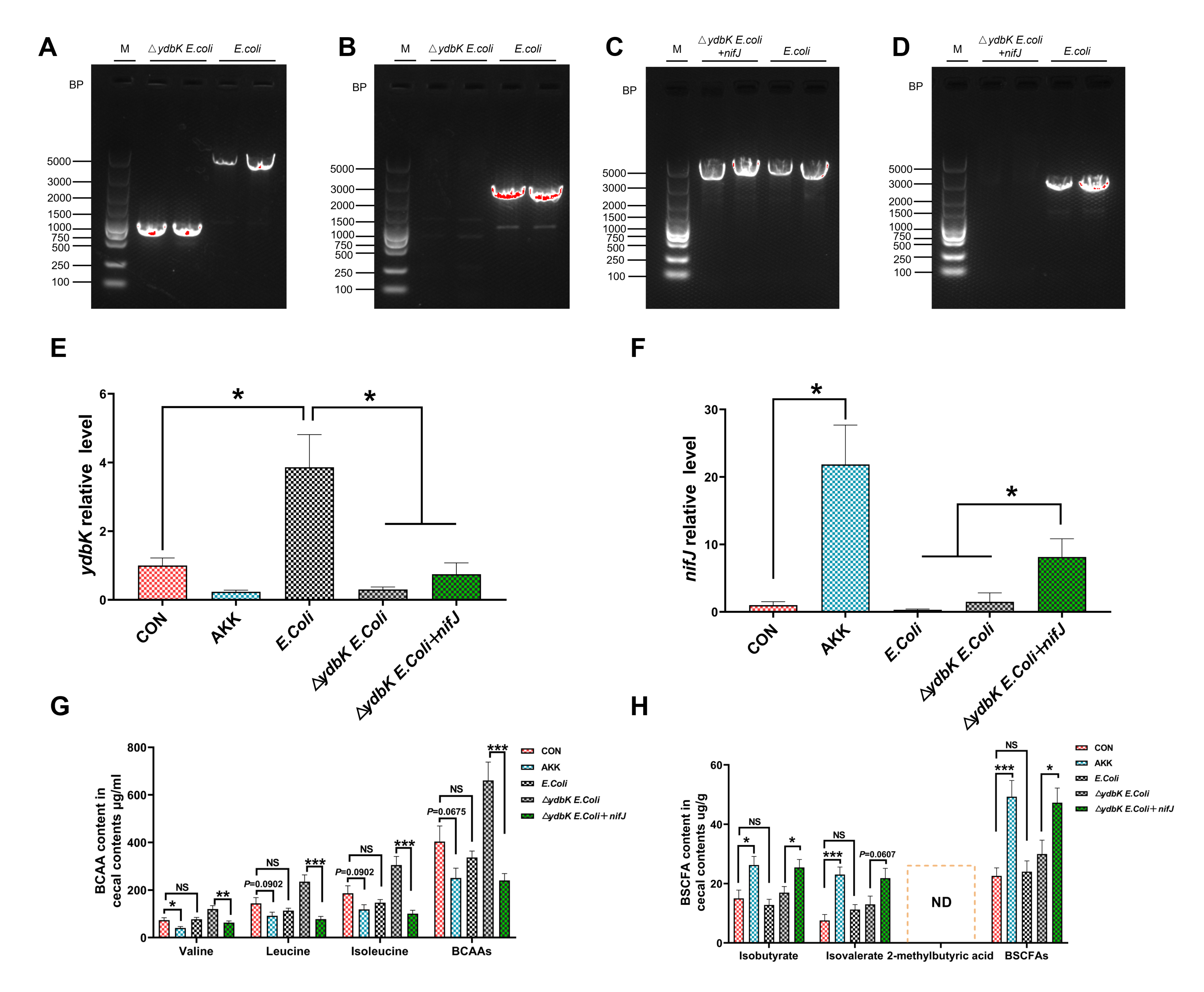


**Supplementary Figure S6. Identification of genetically engineered bacteria and their intestinal colonization in mice.** (A-D) Agarose gel electrophoresis verification of Δ*ydbK* *E. coli* and Δ*ydbK* *E. coli* ＋*nifJ* construction (*n* = 2). (E-F) The *ydbK and nifJ* expression levels in the caecum contents of female mice (*n* = 5 or 6 mice for each group). (G-H) The BCAA and BSCFA content in caecum contents of female mice (*n* = 7 or 8 mice for each group). Δ*ydbK* *E.coli* (A) identification primers are *ydbK*-up-F/*ydbK*-down-R, with the target strain being 813 bp and the original strain being 4338 bp. Δ*ydbK* *E.coli*＋*nifJ* (C) identification primers are *ydbK*-up-F/*ydbK*-down-R, with the target strain being 4543 bp and the original strain being 4338 bp. Δ*ydbK* *E.coli* (B) and Δ*ydbK* *E.coli*＋*nifJ* (D) identification primers are *ydbK*-ter-F/*ydbK*-ter-R, where the target bacteria cannot be amplified, and the original bacteria are 2263 bp. Data in (E-H) are presented as mean ± SEM, and unpaired two-tailed t-test was used to compare two groups. * *P* < 0.05.

| **Supplementary Table S1** Sow body condition and feed intake^1^ | | | |
| --- | --- | --- | --- |
| **Item** | **H-IgA** | **L-IgA** | ***P*-value** |
| **No. of sows** | **25** | **25** |  |
| Body weight, kg |  |  |  |
| G65 d | 226.13±3.97 | 222.28±4.96 | 0.54 |
| L0 d | 219.88±4.58 | 216.98±4.81 | 0.65 |
| Backfat thickness, mm |  |  |  |
| G65 d | 19.72±0.62 | 19.40±0.70 | 0.73 |
| L0 d | 18.20±0.61 | 18.20±0.71 | 1.00 |
| Average daily feed intake, kg/d | 2.24±0.01 | 2.24±0.01 | 0.96 |
| ^1^ All sows entering the trial are first-parity three-way crossbred Duroc-Landrace-Yorkshire sows.  Data are expressed as mean ± SEM. | | | |

**Supplementary Table S2** Primer sequences for qRT-PCR analysis

| **Oligonucleotides** | **Resource** | **Annealing temperature, ℃** |
| --- | --- | --- |
| Primers for mice *18S* RNA | Sangon Biotech | 53.0 |
| Forward: CTTAGTTGGTGGAGCGATTT |  |  |
| Reverse: GCTGAACGCCACTTGTCC |  |  |
| Primers for mice *TGF-β* RNA | Sangon Biotech | 51.0 |
| Forward: ACCGCAACAACGCCATCT |  |  |
| Reverse: GGGCACTGCTTCCCGAAT |  |  |
| Primers for mice *TACI* RNA | Sangon Biotech | 53.0 |
| Forward: CTCAGGAAGGCACCAGGGAT |  |  |
| Reverse: GGCCAAGGCCACCAAGAA |  |  |
| Primers for mice *BAFF* RNA | Sangon Biotech | 49.0 |
| Forward: CTGTTGTCCAGCAGTTTCA |  |  |
| Reverse: TTCGTATAGTCGGCGTGT |  |  |
| Primers for mice *APRIL* RNA | Sangon Biotech | 49.0 |
| Forward: CCTTTCGGTTGCTCTTTG |  |  |
| Reverse: GCTTTGCAGCTCTGTCTGT |  |  |
| Primers for mice *CD40L* RNA | Sangon Biotech | 51.0 |
| Forward: CTCTAATCGGGAGCCTTCG |  |  |
| Reverse: TGGGTATTTGCCGCCTTG |  |  |
| Primers for mice *IL-2* RNA | Sangon Biotech | 47.0 |
| Forward: TGAGTGCCAATTCGATGA |  |  |
| Reverse: AGGGCTTGTTGAGATGATGC |  |  |
| Primers for mice *IL-4* RNA | Sangon Biotech | 49.0 |
| Forward: CATCCTGCTCTTCTTTCTC |  |  |
| Reverse: TTCTCCTGTGACCTCGTT |  |  |
| Primers for mice *IL-5* RNA | Sangon Biotech | 45.0 |
| Forward: GACAAGCAATGAGACGAT |  |  |
| Reverse: AATGACAGGTTTTGGAATA |  |  |
| Primers for mice *IL-6* RNA | Sangon Biotech | 47.0 |
| Forward: TACCACTCCCAACAGACC |  |  |
| Reverse: TTTCCACGATTTCCCAGA |  |  |
| Primers for mice *IL-10* RNA | Sangon Biotech | 45.0 |
| Forward: TTTCAAACAAAGGACCAG |  |  |
| Reverse: GGATCATTTCCGATAAGG |  |  |
| Primers for mice *AID* RNA | Sangon Biotech | 54.2 |
| Forward: TCCTGGAGCCCGTGCTATGAC |  |  |
| Reverse: CTCAGCCTTGCGGTCTTCACAG |  |  |
| Primers for mice *CCL28* RNA | Sangon Biotech | 47.0 |
| Forward: GCTGCTGTCATCCTTCAT |  |  |
| Reverse: TTCCTGCTGGGTTGTTTT |  |  |
| Primers for mice *BCAT1* RNA | Sangon Biotech | 53.0 |
| Forward: TGCCAGCCTCTACATCCG |  |  |
| Reverse: CCAGTCCCACCTTTCCAG |  |  |
| Primers for mice *BCAT2* RNA | Sangon Biotech | 47.0 |
| Forward: ATGAAGGCAAGCAACTCC |  |  |
| Reverse: ACACCCGAAACATCCAAT |  |  |
| Primers for mice *BCKDHA* RNA | Sangon Biotech | 51.0 |
| Forward: CACTACGGCTGCAAGGAA |  |  |
| Reverse: CAGAAGAAGATGATGGGACACT |  |  |
| Primers for mice *BCKDHB* RNA | Sangon Biotech | 45.0 |
| Forward: TATTGCGGAAATCCAGTT |  |  |
| Reverse: GGCTCCCACAGTTGAAAA |  |  |
| Primers for mice *MCT1* RNA | Sangon Biotech | 54.1 |
| Forward: GCCAACACCAAGTGGATCAGACC |  |  |
| Reverse: CGGCGTAGACACAGAACCCAAC |  |  |
| Primers for mice *SMCT1* RNA | Sangon Biotech | 54.2 |
| Forward: TGCTCATCTCGGCGGCTATAGG |  |  |
| Reverse: CACAGGCACGGCGGACATAC |  |  |
| Primers for mice *GPR41* RNA | Sangon Biotech | 52.3 |
| Forward: TGCTCAGAACTTGTGTGCCTTGG |  |  |
| Reverse: AGCTCGGACACTCCTTGGATGG |  |  |
| Primers for mice *GPR43* RNA | Sangon Biotech | 54.2 |
| Forward: GCTGACAGGCTTCGGCTTCTAC |  |  |
| Reverse: GCCACCAGAGCAGCGATCAC |  |  |
| Primers for mice *GPR109A* RNA | Sangon Biotech | 54.1 |
| Forward: GATCATCTGCCTGCCGTTCCTG |  |  |
| Reverse: GATGCTGCCCTGTCGGTTCATAG |  |  |
| Bacterial *16S rRNA* universal primers | Sangon Biotech | 49.0 |
| 1492F: GGTTACCTTGTTACGACTT |  |  |
| 27R: AGAGTTTGATCCTGGCTCAG |  |  |
| *A.muciniphila* 16S rRNA specific primers | Sangon Biotech | 51.0 |
| Forward: GATAGCCCTGGGAAACTG |  |  |
| Reverse: GTAGGTGTCTGGACCGTGT |  |  |
| Primers for *A.muciniphila* *nifJ* RNA | Sangon Biotech | 51.0 |
| Forward: CGGACTTCGCCGCTATTT |  |  |
| Reverse: CGCAGAACGGGAGCATCA |  |  |
| Primers for *E.coli* MG1655 *ydbK* RNA | Sangon Biotech | 49.0 |
| Forward: TCGCCATCTACCCTATTACC |  |  |
| Reverse: TCATCAGCAGCAAACCCT |  |  |

**Supplementary Table S3** Antibodies for Western blotting

| **Antibody** | **Source** | **Identifier** |
| --- | --- | --- |
| IgA | Proteintech | 11449-1-AP, WB dilution 1: 1000, IF dilution 1: 200 |
| TGF-β | ABclonal | A16640, dilution 1: 1000 |
| CCL28 | Proteintech | 18214-1-AP, dilution 1: 800 |
| GPR41 | ABclonal | A12636, dilution 1: 1000 |
| GPR43 | Proteintech | 19952-1-AP, dilution 1: 800 |
| β-actin | ABclonal | AC026, dilution 1: 5000 |

**Supplementary Table S4** Primers used in construction of genetically engineered bacteria

| **Oligonucleotides** | **Source** | **Annealing temperature, ℃** |
| --- | --- | --- |
| Primers for *ydbK* sgRNA | Sangon Biotech | 53.0 |
| TATTGATTTCGTGGGACGTG |  |  |
| Primers for *ydbK*-down | Sangon Biotech | 50.5 |
| F: ATGTAAGGGTGTCATATGCTCTGGATAAGGATTATCCAAT |  |  |
| R: CGAGCAGGGCAGGAATTAAAAGT |  |  |
| Primers for *ydbK*-up | Sangon Biotech | 52.3 |
| F: GCTTCTCCACTACTGGACATCGA |  |  |
| R: ATCCTTATCCAGAGCATATGACACCCTTACATTGCGCAAA |  |  |
| Primers for *ydbK*-pUC19 | Sangon Biotech | 59.7 |
| F: CTATGACCATGATTACGCCGCTTCTCCACTACTGGACATCGA |  |  |
| R: TGTAAAACGACGGCCAGTCGAGCAGGGCAGGAATTAAAAGT |  |  |
| Primers for *ydbK*-ter | Sangon Biotech | 53.0 |
| F: TAATCGGTGTTGCTTTTTTCC |  |  |
| R: TGTTTTATGGCCTTGGTAGTG |  |  |
| Primers for pGEN-*nifJ* | Sangon Biotech | 57.8 |
| F: ACACCACCACCACCACCACTAATGACCTGCAGGCATGCAAGCTTG |  |  |
| R: GGTTGTCTGATCACTCATACGTATCCTCCAAGCCTGAATT |  |  |
| Primers for *nifJ*-pGEN | Sangon Biotech | 58.9 |
| F: GTGGTGGTGGTGGTGGTGTTCCGCAGGTTTGTCGGCACCC |  |  |
| R: CAGGCTTGGAGGATACGTATGAGTGATCAGACAACCGTAA |  |  |
| Primers for *ydbK-*his | Sangon Biotech | 60.2 |
| F: CACCACCACCACCACCACTAAGCTCTGGATAAGGATTATCCAA |  |  |
| Primers for *ydbK-nifJ* | Sangon Biotech | 53.6 |
| R: TGATTAATTGTCAACTATGACACCCTTACATTGCGCAAAT |  |  |
| Primers for *nifJ-ydbK* | Sangon Biotech | 53.6 |
| F: AATGTAAGGGTGTCATAGTTGACAATTAATCATCGGCATA |  |  |
